# Phosphoregulation of the yeast Pma1 H^+^-ATPase autoinhibitory domain involves the Ptk1/2 kinases and the Glc7 PP1 phosphatase and is under TORC1 control

**DOI:** 10.1371/journal.pgen.1011121

**Published:** 2024-01-16

**Authors:** Nadia Guarini, Elie Saliba, Bruno André

**Affiliations:** Molecular Physiology of the Cell, Université Libre de Bruxelles (ULB), Biopark, Gosselies, Belgium; SUNY Upstate Medical University: State University of New York Upstate Medical University, UNITED STATES

## Abstract

Plasma membrane (PM) H^+^-ATPases of the P-type family are highly conserved in yeast, other fungi, and plants. Their main role is to establish an H^+^ gradient driving active transport of small ions and metabolites across the PM and providing the main component of the PM potential. Furthermore, in both yeast and plant cells, conditions have been described under which active H^+^-ATPases promote activation of TORC1, the rapamycin-sensitive kinase complex controlling cell growth. Fungal and plant PM H^+^-ATPases are self-inhibited by their respective cytosolic carboxyterminal tails unless this domain is phosphorylated at specific residues. In the yeast H^+^-ATPase Pma1, neutralization of this autoinhibitory domain depends mostly on phosphorylation of the adjacent Ser911 and Thr912 residues, but the kinase(s) and phosphatase(s) controlling this tandem phosphorylation remain unknown. In this study, we show that S911-T912 phosphorylation in Pma1 is mediated by the largely redundant Ptk1 and Ptk2 kinase paralogs. Dephosphorylation of S911-T912, as occurs under glucose starvation, is dependent on the Glc7 PP1 phosphatase. Furthermore, proper S911-T912 phosphorylation in Pma1 is required for optimal TORC1 activation upon H^+^ influx coupled amino-acid uptake. We finally show that TORC1 controls S911-T912 phosphorylation in a manner suggesting that activated TORC1 promotes feedback inhibition of Pma1. Our results shed important new light on phosphoregulation of the yeast Pma1 H^+^-ATPase and on its interconnections with TORC1.

## Author summary

In all eukaryotic cells, the plasma membrane contains an abundant pump which catalyzes transport of specific ions at the expense of ATP. These ATPases of the P-type superfamily are crucial, as the ion gradient they establish across the membrane supports active uptake of nutrients and export of waste products of cell metabolism. Fungal and plant cells differ from animal cells in that the ion gradient that energizes their plasma membrane is composed of protons (H^+^) instead of sodium (Na^+^). The H^+^-ATPases establishing this H^+^ gradient are highly conserved and also contribute to regulating a kinase complex called TORC1 (Target of Rapamycin Complex 1), which plays a central role in controlling cell growth. The activity of plant and fungal H^+^-ATPases is regulated by a mechanism of self-inhibition mediated by their cytosol-facing C-terminal extremity. The inhibitory effect of this regulatory domain is neutralized by phosphorylation of specific residues. In the yeast *Saccharomyces cerevisiae*, two adjacent C-terminal residues, Ser911 and Thr912, are particularly important in controlling the activity of the main plasma membrane H^+^-ATPase, known as Pma1. This report describes identification of the long-sought kinases and phosphatases modulating phosphorylation of these two residues and reveals a potential contribution of TORC1 to feedback control of this double modification.

## Introduction

The plasma membrane (PM) of fungal and plant cells contains an abundant, highly conserved H^+^-ATPase of the P-type family. These ATPases function as H^+^ pumps which consume a significant fraction of the cellular ATP to maintain cytosolic pH homeostasis and establish the PM H^+^ gradient. This gradient provides the main component of the PM potential and supports active transport of small ions and metabolites via H^+^-coupled transporters [1–4]. In the yeast *Saccharomyces cerevisiae*, the main PM H^+^-ATPase is encoded by the highly expressed and essential gene *PMA1* [5]. The *PMA2* gene encodes a PM H^+^-ATPase isoform which is functional, though unable to compensate for the lack of Pma1 because its expression is too low [6]. Yeast Pma1, which is viewed as the most abundant PM protein, is not homogeneously distributed through the PM. Instead, it accumulates in microdomains distinct from the eisosome compartments into which other transporters segregate [7,8]. Fungal and plant H^+^-ATPases associate into ring-like hexamers [9–12]. Studies in yeast have shown that Pma1 oligomerizes in a manner dependent on sphingolipids during its trafficking along the secretory pathway [13].

**Fig 1:**
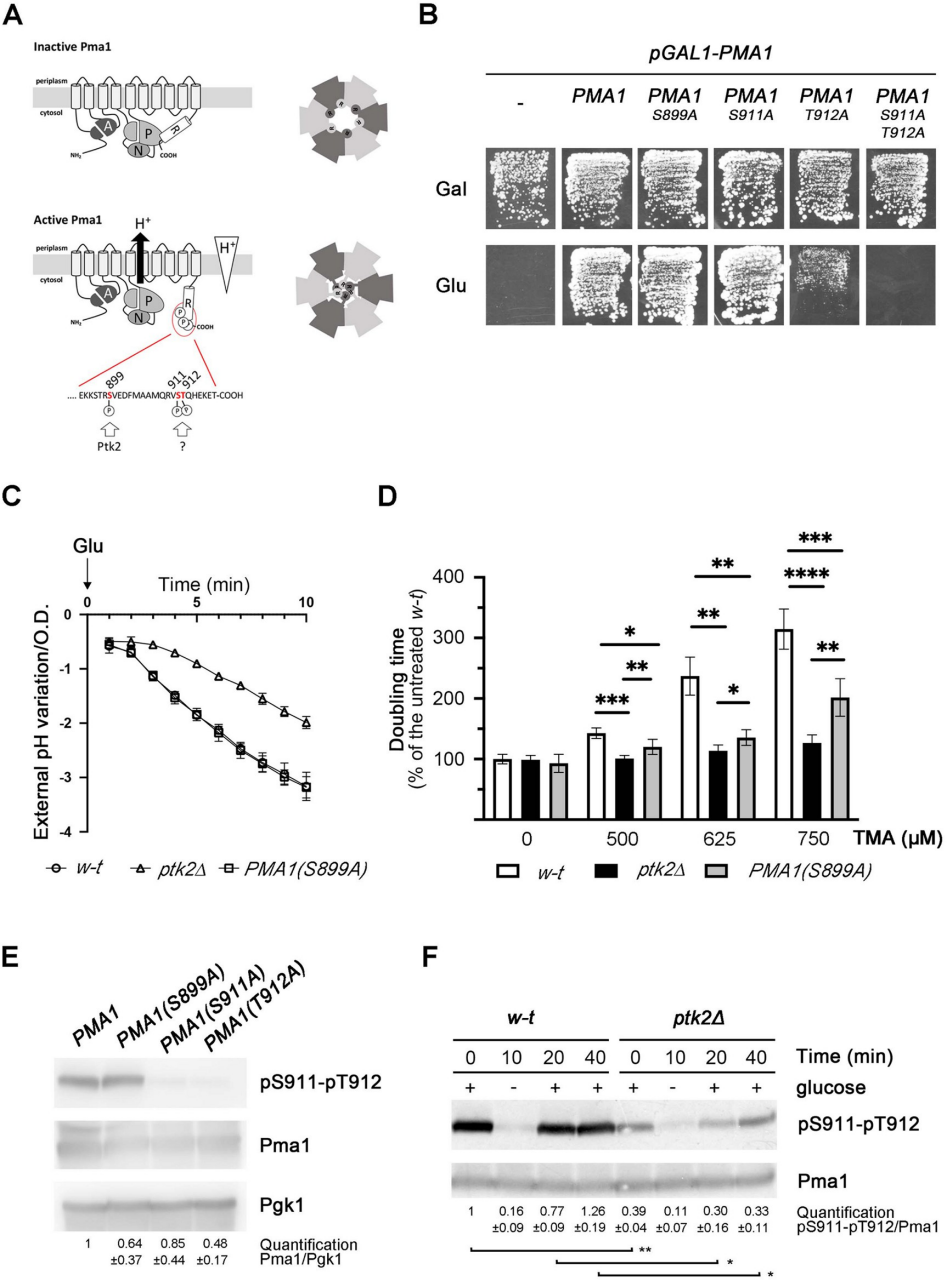
The Ptk2 kinase contributes to tandem S911-T912 phosphorylation in the C-terminal regulatory domain of Pma1. (A) *Left*. Schematic representation of Pma1 self-inhibition by its C-terminal R domain (top) and counteraction of this effect by phosphorylation (bottom). Amino-acid sequence of the Pma1 C-terminus and positions of the S899, S911 and T912 residues. *Right*. Schematic representation of the Pma1 hexamer in its autoinhibited (top) and active (bottom) states. Two shades of gray are used to distinguish adjacent monomers. The different positions of the R domain in active and autoinhibited Pma1 are also schematically represented. (B) *pGAL1-PMA1 pma2Δ* cells expressing from a plasmid the indicated *PMA1* allele were grown for 4 days on solid minimal medium with galactose (Gal) or glucose (Glu) as carbon source. (C) pH variations normalized vs. OD_660_ measured upon glucose addition to glucose-starved *pGAL1-PMA1 pma2Δ* cells expressing *PMA1* (*w-t*) or *PMA1-S899A* from a plasmid and to glucose-starved *pGAL1-PMA1 pma2Δ* *ptk2Δ* cells expressing *PMA1* from a plasmid (*ptk2Δ*). (D) Relative minimal doubling times (% of untreated wild-type) of strains as in C grown on minimal glucose medium supplemented with increasing concentrations of tetramethylammonium (TMA). Bars represent averages ± standard deviation (n = 5), *P* values were calculated using the two-tailed paired *t* test.* *P* < 0,05; ** *P* < 0,01; *** *P* < 0,001; **** *P* < 0,0001. (E) Immunoblot analysis of Pma1 and its phosphorylation at S911-T912 in lysates prepared from *pGAL1-PMA1 pma2Δ* cells growing exponentially in minimal glucose medium and expressing from plasmids the indicated *PMA1* alleles. Total Pma1 was immunodetected with an anti-SpPma1 antibody and Pgk1 was used as an additional loading control. (F) Immunoblot analysis of Pma1 and its phosphorylation at S911-T912 in lysates prepared from *pGAL1-PMA1 pma2Δ* (*w-t*) and *pGAL1-PMA1 pma2Δ* *ptk2Δ* mutant cells expressing Pma1 from a plasmid. Cells were initially grown exponentially in minimal glucose medium (+), transferred for 10 minutes to glucose-free medium (-), and replenished with glucose for 10 or 30 min. * *P* < 0,05; ** *P* < 0,01 (n = 2).

Like all other P-type ATPases, H^+^-ATPase monomers are composed of 10 transmembrane helices and three cytosolic domains, namely the A (actuator), N (nucleotide-binding), and P (phosphorylation) domains (Fig 1A). The cytosolic C-terminal tail is a regulatory (R) domain that can self-inhibit the activity of the H^+^ pump by interacting with the P domain. This autoinhibition is itself regulated by phosphorylation of specific residues in the R domain (Fig 1A) [2,14]. Recent works based on electron cryo-microscopy have established the structures of Pma1 hexamers purified from *S*. *cerevisiae* [11] and *Neurospora crassa* [12]. These analyses revealed that the R domain forms an α-helix which interacts both with the P domain of the same subunit and with that of the neighboring subunit (Fig 1A), thereby reinforcing its clamping effect. Phosphorylation of specific residues in the R domain is predicted to break both cis and trans interactions, thus relieving the clamping effect and enabling P-domain movements coupled to H^+^ export catalysis [11,12]. The untethered R domains of active H^+^-ATPases are proposed to assemble in the central cavity of the hexamer [12] (Fig 1A). As in fungi, the activity of plant H^+^-ATPases is mainly regulated by the C-terminal region, acting as a self-inhibitory domain [2,4]. The effect of this domain is likewise controlled by phosphorylation of specific residues, the most important of which is a Thr at the penultimate position [15,16]. This domain, however, is largely dissimilar in sequence to and larger in size than the R domain of fungal H^+^-ATPases. Furthermore, activation of plant H^+^-ATPases involves association of the phosphorylated R domain with 14-3-3 proteins, which also promote hexamer formation [10,15,17]. Importantly, it is now established that practically all physiological signals regulating plant growth also control the activity of plant H^+^-ATPases via the R domain [2,18].

We have previously reported that yeast and plant PM H^+^-ATPases contribute to regulating TORC1 (Target of Rapamycin Complex 1) [19–21], a kinase complex highly conserved in all eukaryotes and which plays a pivotal role in coordinating cell growth and metabolism [22–24]. The activity of TORC1 is typically low under nutrient deprivation and when any other environmental condition is not favorable to cell growth [22–24]. For instance, yeast TORC1 activity is low in nitrogen-deprived cells, the kinase complex being rapidly reactivated upon active uptake of amino acids or NH_4_^+^ ions [25]. This activation can be decomposed into at least two successive phases relying on different signals and mechanisms [25]. Early activation triggered in the first minutes is transient and dependent on the heterodimeric small GTPase complex (Gtr1/Gtr2) homologous to the RAG GTPase complex of mammalian cells [25,26]. We have reported that the actual signal of this initial TORC1 activation is the influx of H^+^ coupled to active nutrient uptake catalyzed by H^+^ symporters. Furthermore, this H^+^ influx by itself is not sufficient for TORC1 activation, as Pma1 also plays an active role in this process [19]. According to our current model, Pma1 activated by a local increase in cytosolic H^+^ [14] would stimulate an unknown pathway culminating in Gtr1/2-dependent TORC1 activation. Further study on *Nicotiana tabacum* suspension cells has revealed that plant PM H^+^-ATPases likewise promote TORC1 activation. In particular, stimulation of H^+^-ATPases by fusicoccin, a fungal toxin intercalating between their R domain and 14-3-3 proteins [27,28], provokes TORC1 activation. This response is also observed when nutrients and growth hormones are unavailable in the external medium. This suggests that stimulation of the H^+^-ATPase is the trigger of TORC1 activation [21]. The high similarity of yeast and plant PM H^+^-ATPases thus extends also to their ability to control TORC1.

The activity of fungal and plant PM H^+^-ATPases is mainly regulated by phosphorylation of the R domain [4,14,18]. Recent studies have identified the TransMembrane Kinases (TMKs) as the long-sought proteins phosphorylating the penultimate Thr in the R domain of plant H^+^-ATPases in response to auxin [29,30]. In the R domain of yeast Pma1, at least three residues, when phosphorylated, contribute to activating the H^+^-ATPase by destabilizing the inhibitory R-P domain interactions (Fig 1A). The role of these phosphorylations has been studied mostly in the context of Pma1 stimulation in response to high glucose availability. This stimulation coincides with an increase in affinity and Vmax, an optimal pH shifted to more neutral values, and an improved coupling [31,32]. Phosphorylation of one of the R domain residues, Ser899, increases in response to glucose and correlates with higher affinity of Pma1 for ATP [33–35]. It requires the plasma-membrane-associated Ptk2 kinase [34,36], member of a subfamily of yeast Ser/Thr kinases which also includes the TORC1-regulated Npr1 kinase involved in controlling amino acid and NH_4_^+^ permeases [37,38]. Phosphorylation of Ser899 is predicted mostly to weaken R-P interactions between neighboring subunits and to promote association of untethered R domains in the active Pma1 hexamer [11,12]. Phosphorylation of Ser911 and Thr912, two adjacent residues in the R domain of Pma1, also increases upon activation of Pma1 by glucose [39]. This double phosphorylation interferes with both intra- and intersubunit P-R interactions [11,12] and results in an increase of the Vmax of Pma1 [33,40]. Phosphorylation of S911-T912 residues seems crucial for Pma1 activation, as the S911A-T912A double substitution causes strong reduction of Pma1 activity, incompatible with cell growth [41]. In contrast, neither loss of Ptk2 kinase nor the single substitution S899A in Pma1 significantly hampers cell growth [33,36]. The same is true if cells lack Hrk1, another kinase of the Npr1 subfamily required for full Pma1 activity [36] and reported to contribute to S911-T912 phosphorylation, at least under acidic conditions [42]. Hence, although phosphorylation of Ser911 and Thr912 in the R domain plays a central role in Pma1 control, the main kinase(s) responsible for this modification remain uncharacterized, despite several attempts to identify them [34,43].

In this study we reveal that tandem S911-T912 phosphorylation in Pma1 is mediated by the largely redundant Ptk1 and Ptk2 kinases. We further show that dephosphorylation of these residues under glucose starvation requires the Glc7 PP1 phosphatase but not its Reg1 subunit. We also show that the R domain of Pma1 is not required for Pma1-mediated TORC1 activation upon H^+^ influx but can negatively interfere with it when the domain is not fully phosphorylated via the Ptk kinases. We lastly provide evidence that Ptk-dependent phosphorylation of S911-T912 is under TORC1 control, which suggests that TORC1 might feedback-control Pma1 activity.

## Results

### The Ptk2 kinase contributes to tandem S911-T912 phosphorylation in the C-terminal regulatory domain of Pma1

Autoinhibition of yeast Pma1 via its C-terminal regulatory (R) domain is relieved by phosphorylation of Ser899, Ser911, and Thr912 (Fig 1A) [14,34]. Tandem phosphorylation of S911 and T912 is particularly important in counteracting this autoinhibition, as the S911A-T912A double substitution causes strong reduction of Pma1 activity, incompatible with cell growth [41]. This phenotype can be suppressed by removing the R domain or by certain single amino-acid substitutions, some of which alter a P-domain region recently shown to include major sites of contact with the R domain [11,12,33,41]. In contrast, Pma1 variants with a single S899A, S911A, or T912A substitution are reported to remain at least partially active [33,41]. We first ascertained these conclusions by examining growth of a strain in which the chromosomal *PMA1* gene was placed under the control of the galactose-inducible, glucose-repressible *GAL1* promoter. This strain typically fails to grow on glucose unless it contains a plasmid-borne gene expressing an active PM H^+^-ATPase. Normal growth on glucose was restored when the strain expressed Pma1, Pma1 (S899A), or Pma1(S911A) (Fig 1B). In contrast, Pma1(T912A) cells grew much more slowly on this medium and cells expressing Pma1(S911A-T912A) displayed no detectable growth (Fig 1B), in keeping with previous observations [33,41]. This last result illustrates the crucial role of S911-T912 phosphorylation in Pma1 activation, but the main kinase(s) responsible for this double phosphorylation remain unknown.

It has been reported that the Ptk2 kinase is essential to phosphorylation of Ser899 and that this modification confers an increase in the affinity of Pma1 for ATP [33,34,36]. In support of a positive role of Ptk2 in controlling the activity of Pma1, the acidification of the external medium typically observed when glucose-starved cells are replenished with glucose (Fig 1C) was less pronounced in the *ptk2Δ* mutant, as previously reported [36]. However, the strain expressing Pma1(S899A) unexpectedly behaved like the wild type in this assay (Fig 1C). To further compare the phenotypes of *ptk2Δ* and *PMA1(S899A)* cells, we examined their growth rates in the presence of increasing concentrations of tetramethylammonium (TMA) (Fig 1D). Uptake of this toxic cation is typically reduced when the PM potential is diminished, as in mutants with reduced Pma1 activity. The *ptk2Δ* mutant displayed significant resistance to the toxic compound, as expected from a previous report [36]. Although the *PMA1(S899A)* strain also displayed some resistance to TMA, its phenotype was less pronounced than that of the *ptk2Δ* strain (Fig 1D). From these results, we hypothesized that the Ptk2 kinase might promote Pma1 activity by phosphorylating other residues in addition to S899 and that this phosphorylation could be important for activation of Pma1 by glucose.

To analyze a potential role of Ptk2 in phosphorylating residues Ser911 and Thr912, we used an antibody (kindly provided by F. Portillo) raised against a peptide corresponding to the C-terminal residues 904–917 of Pma1 and in which both Ser911 and Thr912 were phosphorylated [34]. In keeping with previous observations [34], this antibody detected a signal in protein extracts prepared from glucose-grown cells expressing Pma1 or Pma1(S899A) (Fig 1E). This signal was almost undetectable in cells expressing the Pma1(S911A) or Pma1(T912A) variant [34] (Fig 1E). Furthermore, the signal intensity decreased markedly upon glucose starvation, resuming rapidly when glucose was re-added to the cultures (Fig 1F). These results corroborate previous observations [34] and are also consistent with those obtained in phosphoproteomic analyses [39,44]. The antibody, which thus specifically recognizes Pma1 doubly phosphorylated at Ser911 and Thr912 [34], also detected a signal in extracts of the *ptk2Δ* mutant. The intensity of this signal was lower than with the wild type, although the observed reduction was mild (Fig 1F). Furthermore, while glucose starvation caused a marked reduction of the signal in the *ptk2Δ* mutant, this mutant showed, upon glucose re-addition, markedly delayed S911-T912 re-phosphorylation as compared to the wild-type (Fig 1F). We conclude that Ptk2 contributes significantly to glucose-regulated tandem phosphorylation of S911-T912 in Pma1.

### The Ptk1 and Ptk2 kinases promote cell viability by counteracting the self-inhibitory domain of Pma1

Although Pma1 phosphorylation at S911-T912 is significantly reduced in the *ptk2Δ* mutant, it remains relatively high (Fig 1F). This indicates that at least one additional kinase contributes with Ptk2 to this modification. The Hrk1 kinase is required for full Pma1 activity [36]. Furthermore, a phosphoproteomic study reported that in cells grown on a glucose medium at pH 4, Hrk1 contributes to basal phosphorylation of a C-terminal peptide of Pma1 and to increase of this phosphorylation upon treatment with acetate [42]. We thus analyzed the influence of Hrk1 on glucose-dependent tandem phosphorylation of S911-T912 (S1A Fig). Mutant cells lacking Hrk1 behaved like the wild-type and the lower S911-T912 phosphorylation in the *ptk2Δ* mutant was not significantly further reduced in the double *ptk2Δ hrk1Δ* mutant strain. Hence, under the conditions of our experiments, Hrk1 does not importantly contribute to glucose-dependent S911-T912 phosphorylation in Pma1.

**Fig 2:**
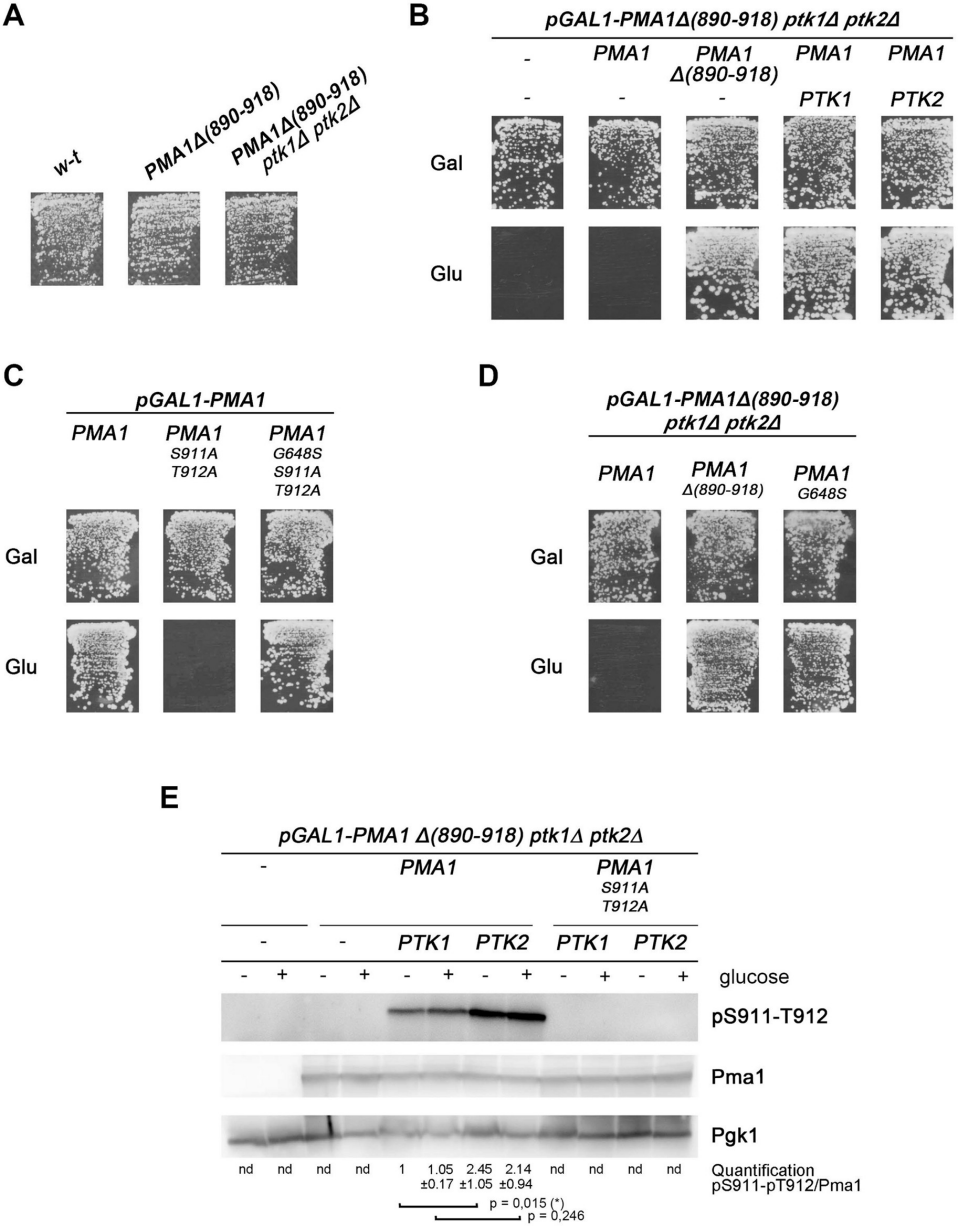
The Ptk1 and Ptk2 kinases promote cell viability by counteracting the C-terminal self-inhibitory domain of Pma1. (A) Strains with the indicated genotypes were grown for 3 days on solid minimal glucose medium. (B) *pGAL1-PMA1Δ(890–918) pma2Δ* *ptk1Δ* *ptk2Δ* cells expressing from plasmids the indicated *PMA1* allele and *PTK* gene(s) were grown for 4 days on solid minimal medium containing galactose (Gal) or glucose (Glu) as carbon source. (C) *pGAL1-PMA1 pma2Δ* cells expressing from a plasmid the indicated *PMA1* allele were grown for 3 days on solid minimal glucose medium. (D) *pGAL1-PMA1Δ(890–918) pma2Δ* *ptk1Δ* *ptk2Δ* cells expressing from a plasmid the indicated *PMA1* allele were grown for 3 days on a solid minimal glucose medium. (E) Immunoblot analysis of Pma1 and its phosphorylation at S911-T912 in lysates prepared from cells growing exponentially on minimal galactose medium before (-) and 30 min after (+) glucose addition. The strains were *pGAL1-PMA1Δ(890–918) pma2Δ* *ptk1Δ* *ptk2Δ* expressing from plasmids the indicated *PMA1* allele and *PTK* gene. Pgk1 was included as an additional loading control for cell lysates where no Pma1 signal was detectable. * *P* < 0,05 (n = 5).

The *PTK2* gene has a paralog, *PTK1*, which most likely arose through whole genome duplication in a yeast ancestor [45]. A strain with deletion of the *PTK1* gene was found to acidify the external medium in response to glucose as efficiently as the wild type (S2A Fig). Furthermore, in the *ptk1Δ* mutant grown on glucose, S911-T912 phosphorylation in Pma1 seemed normal, and after its rapid drop upon glucose starvation, it resumed normally when the cells were replenished with glucose (S2B Fig). These results indicate that loss of Ptk1 does not significantly impede the glucose-dependent activity of Pma1 nor its phosphorylation at S911-T912. We also analyzed sensitivity of the *ptk1Δ* strain to TMA but did not find pronounced resistance as with the *ptk2Δ* strain (S2C Fig). We next crossed the *ptk1Δ* and *ptk2Δ* strains to isolate a *ptk1Δ ptk2Δ* double mutant. Importantly, this turned out to be unfeasible, as *ptk1Δ ptk2Δ* haploids were not viable (S3 Fig). Lack of growth is also the phenotype displayed by cells expressing the Pma1(S911A-T912A) mutant [41] (Fig 1B). The synthetic lethality of the *ptk1Δ ptk2Δ* mutant thus suggests that Ptk1 and Ptk2 might redundantly promote S911-T912 phosphorylation in Pma1 to neutralize the self-inhibitory R domain. If this is true, deletion of this domain in Pma1 should restore high activity of the H^+^-ATPase in the *ptk1Δ ptk2Δ* mutant, which should thus also recover viability. This was assessed first by applying CRISPR-Cas9 to the *ptk2Δ* mutant to introduce into the 3’ end of the *PMA1* gene a deletion giving rise to a truncated Pma1 lacking the C-terminal residues 890–918. Crossing the resulting *ptk2Δ PMA1(Δ890–918)* strain with the *ptk1Δ* mutant resulted in isolation of many viable haploid segregants, some of which did display the *ptk1Δ ptk2Δ PMA1(Δ890–918)* genotype. Furthermore, the growth of these strains on solid medium was similar to that of the wild-type and *PMA1(Δ890–918)* strains (Fig 2A). Taken together, these results show that the yeast Ptk1 and Ptk2 kinases act in a largely redundant manner to neutralize the autoinhibitory domain of Pma1, a function crucial for cell growth.

The essential role of Ptk1/2 in stimulating Pma1 activity was ascertained by isolating a *ptk1Δ ptk2Δ* mutant where the chromosomal *PMA1* gene was placed under the control of the *GAL1* promoter and its 3’-end was deleted so as to eliminate residues 890–918 of the H^+^-ATPase. This mutant was able to grow on galactose but not on glucose, because no Pma1 protein was produced on the latter medium (Fig 2B). The same phenotype was observed after introduction of a plasmid expressing the *PMA1* gene under its own promoter. This was expected, as no functional Ptk kinase was present. In contrast, the strain recovered growth on glucose when it expressed the truncated Pma1(Δ890–918). Importantly, normal growth on glucose was also observed when the cells expressed the full-length Pma1 together with Ptk1 or Ptk2 (Fig 2B). These results confirm that Pma1, when possessing its autoinhibitory R domain, is not functional in cells lacking both Ptk kinases. Furthermore, expression of Ptk1 or Ptk2 alone is sufficient to restore Pma1 functionality and support growth.

According to a previous report, a strain expressing the poorly active Pma1(S911A -T912A) mutant recovers high H^+^-ATPase activity and growth if the mutant Pma1 contains an additional G648S substitution [33]. We confirmed this in a growth assay (Fig 2C). The G648 residue lies in a region of the cytosolic P domain of Pma1 that is close to several sites of contact with the R domain [11,12]. This suggests that G648S interferes with the self-inhibition mediated by the unphosphorylated C-tail of Pma1. Interestingly, the Pma1(G648S) variant also proved able to restore growth on glucose of the strain lacking both Ptk1 and Ptk2 kinases (Fig 2D). This result confirms that in glucose-growing cells, Ptk1 and Ptk2 act in a largely redundant manner to prevent the R domain of Pma1 from exerting its self-inhibitory effect.

### Ptk1 and Ptk2 mediate tandem phosphorylation of S911-T912 in Pma1

We next examined the roles of the Ptk1 and Ptk2 kinases in S911-T912 tandem phosphorylation of Pma1. The experiment exploited the *pGAL1-PMA1(Δ890–918) ptk1Δ ptk2Δ* strain, which can grow on galactose as a carbon source thanks to induced genomic expression of the truncated Pma1(Δ890–918). We transformed the strain with a plasmid expressing the *PMA1* gene under its own promoter. As this gene is naturally expressed in cells using galactose as a carbon source, the transformed strain growing on galactose medium should co-express two Pma1 proteins: the active, C-terminally truncated Pma1(Δ890–918) from the genome and the full-length Pma1 protein from the plasmid. The latter should not be active, as neither Ptk1 nor Ptk2 is present to phosphorylate it at S911 and T912. In support of this view, an antibody raised against the C-terminal tail of Pma1 [46] detected, in cell lysates of this strain, a signal corresponding to the plasmid-encoded, full-length Pma1 (Fig 2E). This Pma1 protein, however, was not detected with the antibody recognizing the protein doubly phosphorylated at S911-T912 (Fig 2E). In contrast, the latter antibody gave rise to a signal if the strain additionally expressed the Ptk1 or Ptk2 kinase. No signal was observed with this antibody, however, when cells expressing Ptk1 or Ptk2 together with the Pma1(S911A-T912A) variant were used (Fig 2E). These results confirm that both Ptk1 and Ptk2 mediate tandem S911-T912 phosphorylation in Pma1. Comparison of the intensity of this phosphorylation in Ptk1- and Ptk2-expressing cells showed that Ptk2 promotes this double phosphorylation more efficiently than Ptk1 (Fig 2E), in agreement with the more pronounced phenotype displayed by the *ptk2Δ* mutant than by the *ptk1Δ* strain. Of note, adding glucose to galactose-grown cells for 30 min did not significantly increase either Ptk1- or Ptk2-dependent S911-T912 phosphorylation (Fig 2E). Furthermore, the apparent more important contribution of Ptk2 in S911-T912 phosphorylation was also observed in cells expressing Pma1(S899A) or Pma1(S899D), indicating that it is not due to Ptk2’s role in S899 phosphorylation which would in turn favor S911-T912 phosphorylation (S4 Fig). In conclusion, these results demonstrate that the largely redundant Ptk1 and Ptk2 kinases mediate phosphorylation of S911-T912 in the C-terminal R domain of Pma1.

### Glc7 phosphatase mediates carbon starvation-induced dephosphorylation of S911-T912 in Pma1

**Fig 3:**
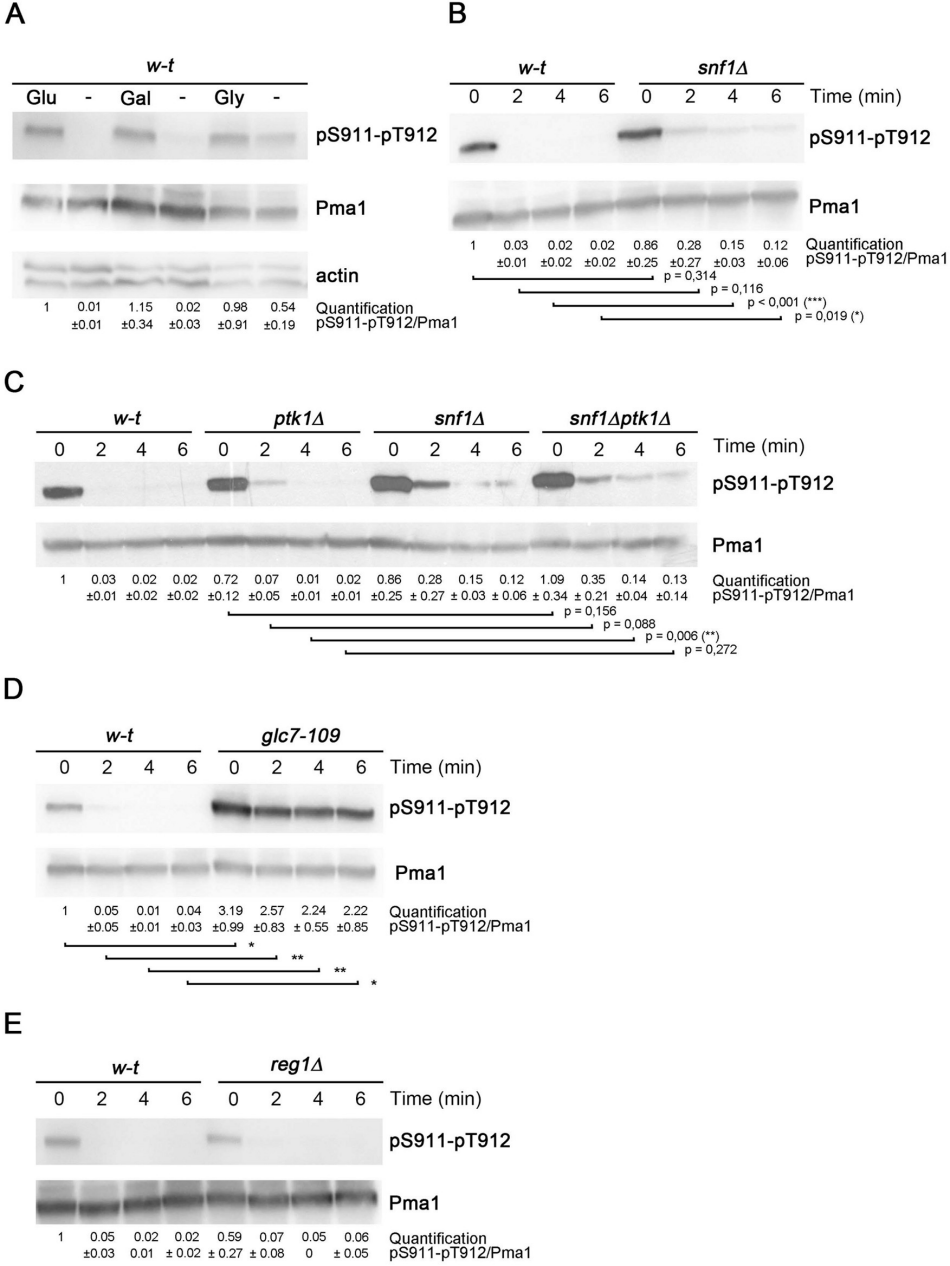
Glc7 phosphatase mediates dephosphorylation of S911-T912 in Pma1 under carbon starvation. (A) Immunoblot analysis of Pma1 and its phosphorylation at S911-T912 in lysates prepared from wild-type cells growing exponentially in minimal medium containing glucose (Glu), galactose (Gal), or glycerol (Gly) as carbon source and transferred for 10 min to the same medium except that no carbon source was available. (B, C, D, E) Immunoblot analysis of Pma1 and its phosphorylation at S911-T912 in lysates prepared from cells collected during exponential growth in minimal glucose medium (0 min) and after transfer for the indicated time to the same medium except that no carbon source was available. * *P* < 0,05; ** *P* < 0,01; *** *P* <0,001 (n = 2 to 4).

Upon glucose starvation, the R domain of Pma1 is rapidly dephosphorylated, allowing it to establish contacts with the P domain of the H^+^-ATPase, thus causing its self-inhibition [34,39]. This coincides with a decrease of both the Vmax of the H^+^ pump and its affinity for ATP, a shift of the optimal pH to more alkaline values, and less efficient coupling [31,32,47]. Furthermore, the intracellular ATP needed to fuel the activity of the H^+^-ATPase’s drops rapidly upon glucose starvation. When cells were pregrown on a galactose medium, a shift to a medium devoid of any carbon source also elicited rapid S911-T912 dephosphorylation in Pma1, equivalent to that observed in cells pre-cultivated on glucose (Fig 3A). This dephosphorylation was less pronounced in cells initially grown on glycerol, a respiratory carbon source (Fig 3A). Of note, a previous phosphoproteomic analysis has revealed that loss of S911-T912 double phosphorylation upon glucose starvation involves, in fact, rapid dephosphorylation of S911 alone followed by slower dephosphorylation of the adjacent T912 [39]. Hence, reduction of the intensity of the immunoblot signal corresponding to double S911-T912 phosphorylation (Fig 3A) might in fact reflect a situation where single T912 phosphorylation subsists for a certain time. We next sought to better characterize the mechanisms underlying the rapid loss of tandem S911-T912 phosphorylation upon carbon starvation. Snf1 kinase, the yeast homolog of mammalian AMPK, is stimulated under glucose starvation [48]. Furthermore, previous phosphoproteomic analyses have indicated that several residues in Ptk2 are hyperphosphorylated via Snf1 in glucose-starved cells [44,49]. Although no equivalent control has been described for Ptk1, this observation prompted us to hypothesize that Snf1 might contribute, via phospho-inhibition of Ptk2, to reducing tandem S911-T912 phosphorylation in Pma1. In the *snf1Δ* mutant starved of glucose, Pma1 still underwent efficient dephosphorylation of the S911-T912 pair, although slight protection against complete dephosphorylation was reproducibly observed (Fig 3B). This mild phenotype was shared by similarly treated *snf1Δ ptk1Δ* mutant cells, where Ptk2 is the only active Ptk kinase (Fig 3C). Hence, Snf1 plays a modest though significant role in reducing tandem S911-T912 phosphorylation in glucose-starved cells, possibly via phosphocontrol of the Ptk2 kinase.

Stimulation of a phosphatase might also contribute to reduced tandem phosphorylation of Pma1 residues S911-T912 in glucose-starved cells. The essential gene *GLC7* encodes a PP1 phosphatase which associates with one of various regulatory subunits and catalyzes dephosphorylation of a wide variety of target proteins [50]. In particular, Glc7 together with the Reg1 subunit dephosphorylates Snf1 under conditions of ample glucose availability [48]. Importantly, investigators have previously reported isolation of a particular mutant allele of *GLC7*, *glc7-109*, exhibiting the traits typically displayed by mutants in which Pma1 is hyperactive, e.g. increased sensitivity to cations and aminoglycosides [51]. Furthermore, mutations suppressing this phenotype were found to affect the *PMA1* gene and to reduce the activity of the H^+^-ATPase [51]. As the particular phenotype of this *glc7-109* mutant differs from those displayed by other viable *glc7* mutant strains, it has been proposed that the Glc7 phosphatase encoded by *glc7-109* is specifically defective in a mechanism of downregulation of Pma1 activity [51]. The phenotype of the *glc7-109* mutant is caused by an R260A substitution affecting an Arg residue located at the surface of the protein. This Arg residue might thus possibly contribute to interaction of Glc7 with proteins involved in Pma1 control or with the H^+^-ATPase itself [51]. Importantly, *glc7-109* was reported to impair glucose starvation-elicited dephosphorylation of S899 in Pma1 [34]. These previous observations prompted us to examine the influence of the *glc7-109* mutation on S911-T912 dephosphorylation induced by glucose deprivation. Remarkably, tandem S911-T912 phosphorylation remained high in *glc7-109* mutant cells transferred to a glucose-free medium, but was rapidly lost in the corresponding wild type (Fig 3D). Basal phosphorylation of S911-T912 was also markedly increased in the *glc7-109* mutant grown on glucose (Fig 3D), indicating that Glc7 moderates Pma1 S911-T912 phosphorylation also under conditions of normal glucose supply. This increased basal phosphorylation likely accounts for the higher activity of Pma1 in the *glc7-109* mutant strain grown on glucose [51].

Reg1 is a well-known subunit of Glc7, required for Glc7-dependent dephosphorylation of multiple proteins, including Snf1, in glucose-fed cells [48]. Interestingly, our results show, rather, that Glc7 promotes Pma1 dephosphorylation upon glucose starvation, conditions under which the Glc7/Reg1 complex is normally less active [48]. We nevertheless examined whether Reg1 might be involved in S911-T912 dephosphorylation in Pma1. In *reg1Δ* cells, loss of tandem S911-T912 phosphorylation upon glucose starvation was as efficient as in wild-type cells (Fig 3E), consistently with the results of another study [34]. We thus hypothesized that Glc7 might function conjointly with another regulatory subunit to dephosphorylate Pma1. To assess this model, we tested the influence of single deletion of 22 genes encoding previously described Glc7 regulatory subunits [50], but found Pma1 to undergo normal S911-T912 dephosphorylation in glucose-starved cells (S5 Fig). It could be that Glc7 associates with either of two (or more) redundant regulatory subunits to promote Pma1 dephosphorylation under glucose starvation.

### Reduced Ptk kinase activity impedes TORC1 activation upon H^+^-coupled amino-acid uptake

**Fig 4:**
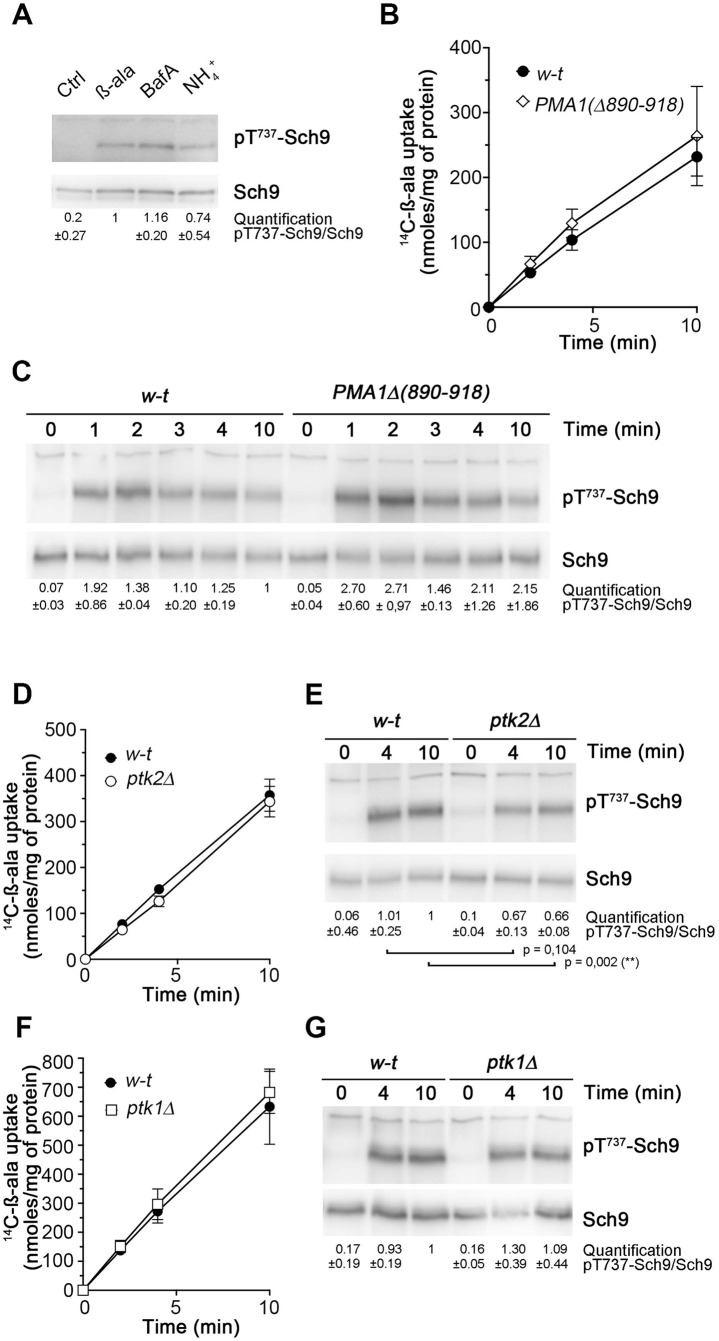
Reduced Ptk kinase activity impedes TORC1 activation upon H^+^-coupled amino-acid uptake. (A) Immunoblot analysis of Sch9 and its phosphorylation at T737 in lysates prepared from wild-type cells growing exponentially on minimal glucose proline medium before (Ctrl) and 4 minutes after treatment with Bafilomycin A (BafA, 1 μM), β-alanine (β-ala, 0.5 mM), or NH_4_^+^ (1 mM). (B) Equivalent uptake of [^14^C]-β-alanine in *pGAL1-PMA1 pma2Δ* cells expressing *PMA1* or *PMA1Δ(890–918)* from a plasmid. The cells were initially grown to exponential phase in glucose NH_4_^+^ medium before being transferred for two hours to the same medium except that it lacked nitrogen. The labeled amino acid was then added (time 0 min) at a final concentration of 0.25 mM (*PMA1* cells) or 0.5 mM (*PMA1Δ(890–918*) cells). (C) Immunoblot analysis of Sch9 and its phosphorylation at T737 in lysates prepared from *pGAL1-PMA1 pma2Δ* cells treated as in B except that β-alanine was unlabeled. (D) Equivalent uptake of [^14^C]-β-alanine in wild-type and *ptk2Δ* mutant cells cultivated and treated as in B. The labeled amino acid was added (time 0 min) at a final concentration of 0.25 and 0.5 mM, respectively (E) Immunoblot analysis of Sch9 and its phosphorylation at T737 in lysates prepared from wild-type and *ptk2Δ* mutant cells treated as in B except that β-alanine was unlabeled. ** *P* < 0,01 (n = 3). (F, G) Same as in D and E except that the analyzed strains corresponded to the wild-type and *ptk1Δ* mutant treated with identical β-alanine concentrations (0.5 mM).

We have previously shown that Pma1 plays an important role in early TORC1 activation upon active uptake of amino acids into nitrogen-starved cells [19]. According to the proposed model, transport of these amino acids via H^+^ symporters causes a local increase in cytosolic H^+^ which stimulates Pma1 activity. This H^+^-stimulated Pma1 would then elicit, via an unknown mechanism, a signaling cascade culminating in TORC1 activation via the heterodimeric Gtr1/2 small GTPase complex [19]. To investigate potential links between the R domain of Pma1 and H^+^-increase-elicited TORC1 activation, we first grew cells on a poor nitrogen source, namely proline. Under these conditions, TORC1 activity is typically low. We then treated the cells with β-alanine, an amino acid whose uptake via the general amino acid permease, Gap1, is coupled to H^+^ influx [19]. Of note, β-alanine cannot be used as a nitrogen source and its uptake into cells does not increase the intracellular concentration of other amino acids [19]. In keeping with previous observations [19], β-alanine transport elicited a rapid increase in phosphorylation at Thr737 in the Sch9 kinase, indicative of increased TORC1 activity (Fig 4A). A similar response was observed after addition of either NH_4_^+^, whose uptake via the Mep1 permease is also coupled to H^+^ co-transport [52], or the V-ATPase inhibitor bafilomycin A, which causes an increase in cytosolic H^+^ [1]. We next sought to determine whether truncating Pma1 of its C-terminal autoinhibitory domain alters H^+^-increase-elicited TORC1 activation. We thus initially grew cells expressing full-length Pma1 or the truncated Pma1(Δ890–918) variant on proline medium before adding β-alanine. For unclear reasons, the effect of β-alanine on TORC1 activity in Pma1(Δ890–918)-expressing cells varied according to experiments, so that no clear conclusion could be drawn from them. We could circumvent this difficulty by first growing cells on NH_4_^+^, a nitrogen source supporting high TORC1 activity, before transferring them for two hours to a nitrogen-free medium to cause TORC1 inhibition. β-Alanine was then added to trigger TORC1 reactivation. Under these conditions, we observed, as expected, a very low initial TORC1 activity which markedly increased upon β-alanine addition (S6A and S6B Fig). This stimulation of TORC1 was impaired in a mutant lacking the Gtr1/2 small GTPases (S6C Fig), in keeping with previous observations [19]. We then applied this experiment to wild-type and Pma1(Δ890–918) cells, after having adjusted the concentrations of β-alanine to ensure equivalent incorporation (Fig 4B). Under these conditions, we observed that the wild-type and *PMA1(Δ890–918)* strains both exhibited efficient TORC1 reactivation upon equivalent β-alanine uptake (Fig 4C). This results thus shows that the C-terminal 890–918 region of Pma1 is not essential to H^+^-increase-elicited TORC1 activation. In contrary, this TORC1 activation seemed higher when the C-terminal tail of Pma1 was truncated, although the difference was mild (Fig 4C).

In the *ptk2Δ* mutant grown on glucose, Pma1 is less active because its C-terminal autoinhibitory domain is not fully neutralized by phosphorylation. We next sought to determine if this partial self-inhibition also impedes the role of Pma1 in TORC1 activation. For this we compared TORC1 activation upon β-alanine uptake in wild-type and *ptk2Δ* mutant cells. The concentration of β-alanine added to wild-type cells was first adjusted to ensure its uptake at the same rate as in *ptk2Δ* cells (Fig 4D). Interestingly, TORC1 activation upon equivalent β-alanine transport was significantly reduced in the *ptk2Δ* mutant (Fig 4E). We also analyzed TORC1 activation in the *ptk1Δ* mutant, where reduction of Pma1 activity is much less pronounced than in the *ptk2Δ* mutant. In this case, no significant reduction of TORC1 activation was detected upon equivalent uptake of β-alanine (Fig 4F and 4G). Finally, TORC1 was normally activated by β-alanine also in the *glc7-109* mutant (S7 Fig). In conclusion, loss of Ptk2 kinase interferes not only with the activity of the H^+^-ATPase but also with TORC1 activation triggered by H^+^ influx. The simplest interpretation of this result is that the R domain of Pma1, when it is not properly phosphorylated via the Ptk kinases, negatively interferes with TORC1 activation promoted by H^+^-stimulated Pma1.

### TORC1 controls S911-T912 phosphorylation in Pma1

**Fig 5:**
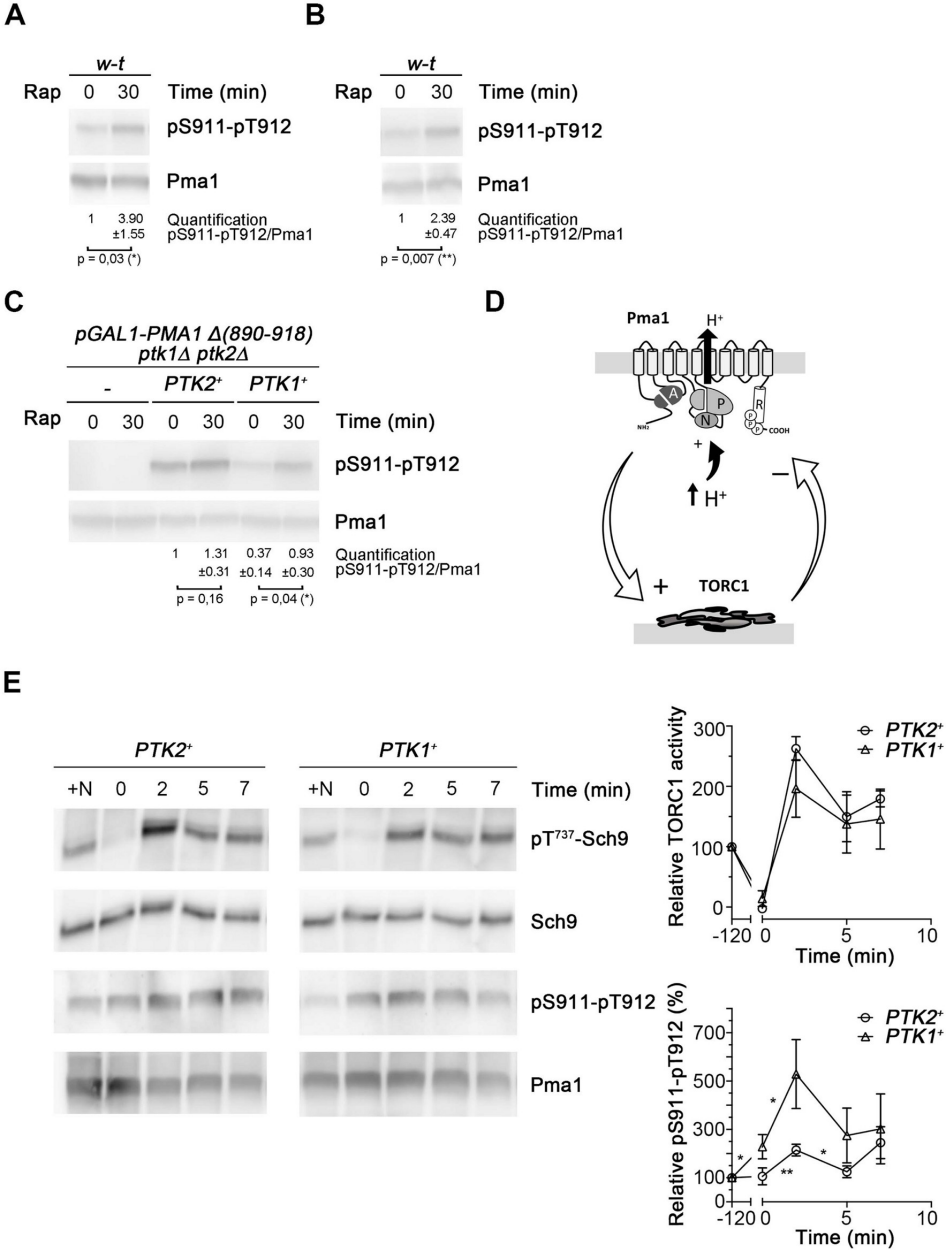
TORC1 controls S911-T912 phosphorylation in Pma1. (A) Left: Immunoblot analysis of Pma1 and its phosphorylation at S911-T912 in lysates prepared from wild-type cells growing exponentially in minimal glucose medium before and after treatment with rapamycin (Rap, 200 ng/ml). * *P* < 0,05 (n = 3). (B) Same as in A except that cells were grown on galactose medium.). ** *P* < 0,01 (n = 3). (C) Same as in A except that cells were *pGAL1-PMA1Δ(890–918) pma2Δ* *ptk1Δ* *ptk2Δ* growing in minimal galactose medium and expressing from plasmids the *PMA1* gene and a single or no (-) *PTK* gene. * *P* < 0,05 (n = 3). (D) Schematic model of TORC1 activation induced upon Pma1 stimulation by H^+^ increase, in turn promoting a reduction of Pma1 activity through downregulation of its S911-T912 phosphorylation. (E). Left: Immunoblot analysis of Pma1 and its phosphorylation at S911-T912 and Sch9 and its phosphorylation at T737 in lysates prepared from *ptk1Δ* (*PTK2*^+^) and *ptk2Δ* (*PTK1*^+^) mutant cells growing exponentially in glucose NH_4_^+^ medium (+N), transferred for 2 hours to nitrogen-free medium (time 0), and incubated for the indicated time with β-alanine (0.5 mM to *ptk2Δ* cells, 0,25 mM to *ptk1Δ* cells for equivalent uptake). Right: Quantifications of T737-Sch9 phosphorylation vs. total Sch9 (top) and of S911-T912 phosphorylation vs. total Pma1 (bottom). The significance of the difference between two successive time points was evaluated using Student’s two-tailed *t*-test * *P* < 0,05; ** *P* < 0,01 (n = 3) (S1 Table).

Phosphorylation of several upstream regulators of yeast TORC1 is reported to be under TORC1 control, allowing their feedback control which in turn fine-tunes TORC1 activity [53–55]. We thus wondered if phosphorylation of S911-T912 in Pma1 might be influenced by TORC1 activity. Interestingly, this view is supported by the data of several phosphoprotemic studies having revealed increased S911-T912 phosphorylation in rapamycin-treated cells [56–58]. In our experiments, we likewise found rapamycin addition to cause a net increase in Pma1 tandem S911-T912 phosphorylation (Fig 5A). To ascertain that this increased phosphorylation was mediated by Ptk1/2 and not via another kinase potentially stimulated upon TORC1 inhibition, we used the *pGAL1-PMA1(Δ890–918) ptk1Δ ptk2Δ* strain expressing full length Pma1 from a plasmid. As this strain can only grow on galactose as a carbon source, we first examined whether wild-type cells grown on this medium also show increased Pma1 S911-T912 phosphorylation upon rapamycin addition. This turned out to be the case (Fig 5B). In contrast, this phosphorylation increase was not detected for Pma1 expressed from a plasmid in the *pGAL1-PMA1(Δ890–918) ptk1Δ ptk2Δ* strain (Fig 5C). This confirms that the increase depends on Ptk1/2. The experiment was also carried out in the same strain co-expressing Pma1 and Ptk1 or Ptk2 from plasmids (Fig 5C). Before rapamycin treatment, both Ptk1 and Ptk2 were found, as expected, to promote S911-T912 phosphorylation, which was lower in Ptk1- than in Ptk2-expressing cells, as previously observed. Interestingly, rapamycin addition caused a more pronounced increase in Pma1 S911-T912 phosphorylation in Ptk1- than in Ptk2-expressing cells (Fig 5C), indicating that TORC1 inhibition mostly stimulates Ptk1-dependent phosphorylation of S911-T912.

The above observations based on analysis of rapamycin-treated cells led us to predict that TORC1, when activated, might downregulate Ptk-dependent phosphoactivation of Pma1, especially that mediated by Ptk1 (Fig 5D). To assess this view, we starved *PTK1*^+^ (*ptk2Δ*) and *PTK2*^+^ (*ptk1Δ*) cells for nitrogen, supplied them with β-alanine, and analyzed in parallel the phosphorylation of T737 in Sch9 and S911-T912 in Pma1 (Fig 5E). A significant increase in S911-T912 phosphorylation was detected upon nitrogen starvation of *PTK1*^+^ cells (*P* = 0, 011, n = 3) but not of *PTK2*^+^ cells (Fig 5E). This is in keeping with the more pronounced response to rapamycin of Ptk1- vs. Ptk2-dependent phosphorylation (Fig 5C). Importantly, addition of β-alanine for two minutes, which induced rapid TORC1 activation as expected (Fig 5E), caused also an increase in S911-T912 phosphorylation via both Ptk1 (*P* = 0.026, n = 3) and Ptk2 (*P* = 0.004, n = 3) (Fig 5E). This effect was rapidly followed by a decrease in S911-T912 phosphorylation detectable five minutes after β-alanine addition (*P* = 0.074 in *PTK1*^+^ cells, P = 0.015 in *PTK2*^+^ cells, n = 3) (Fig 5E). In keeping with the more pronounced effect of rapamycin on Pma1 phosphorylation mediated by Ptk1 vs. Ptk2 (Fig 5C), these effects of β-alanine seemed more pronounced in *PTK1*^+^ cells (Fig 5E). In conclusion, these results indicate that H^+^-coupled amino-acid uptake promotes an increase followed by a decrease in both S911-T912 phosphorylation and TORC1 activity. The decrease in S911-T912 phosphorylation observed after TORC1 activation supports the proposed model that TORC1 promotes feedback-inhibition of Pma1 phosphorylation at S911-T912 (Fig 5D).

## Discussion

**Fig 6:**
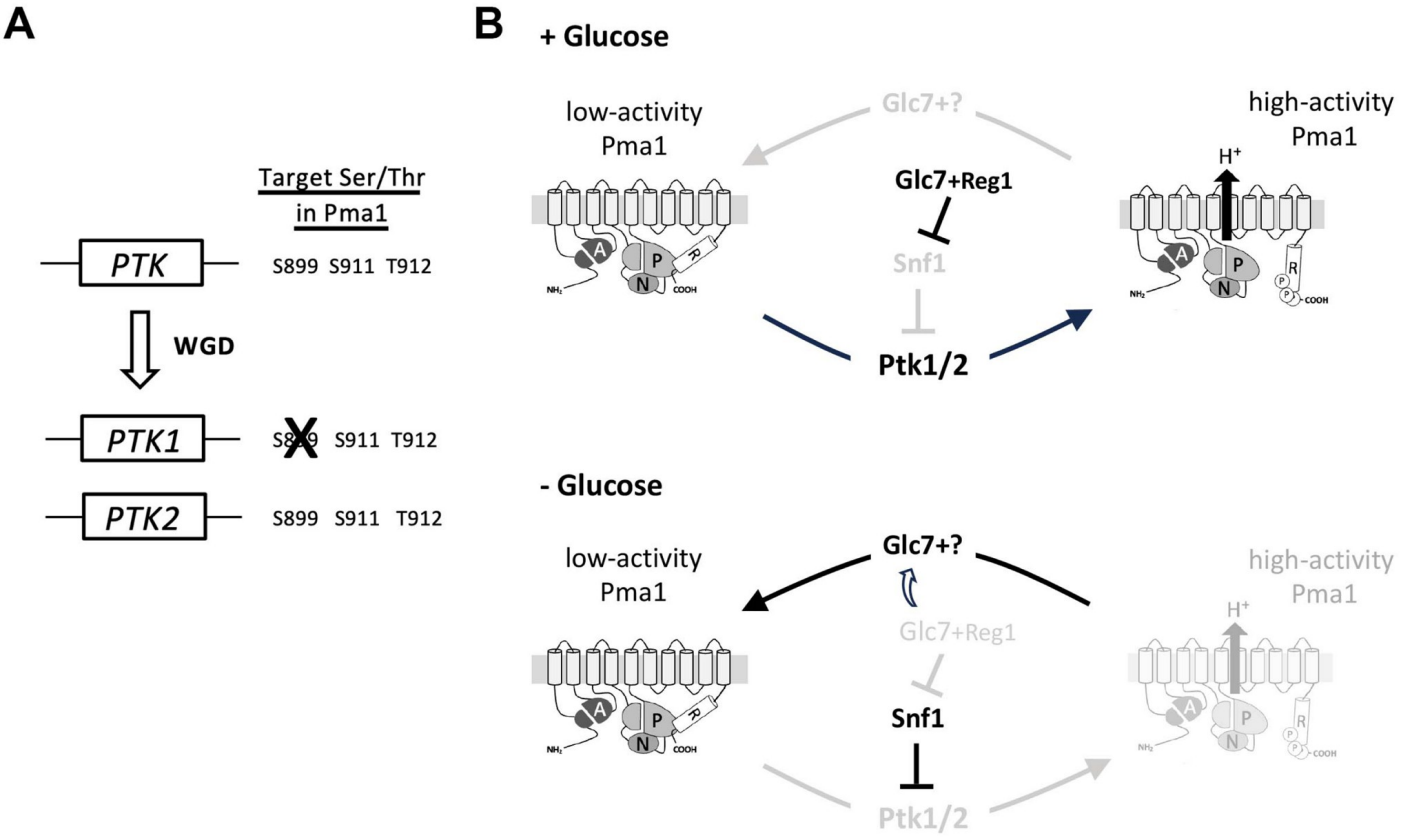
Evolution and role in Pma1 regulation of Ptk kinases. (A) Hypothetical model of *PTK* genes evolution (see text). WGD: whole genome duplication. (B) Proposed model of Pma1 regulation by phosphorylation of its C-terminal R domain. In the presence of glucose, Glc7/Reg1 is active and inhibits Snf1. In parallel, the Ptk1 and Ptk2 kinases mediate phosphorylation of S899, S911, and T912 in the R domain of Pma1. This disrupts the self-inhibitory interactions between the R and P domains of Pma1, which is thus active. Under glucose starvation, Glc7/Reg1 is less active and this promotes stimulation of Snf1, which contributes to inhibition of the Ptk kinases. In parallel, the Glc7 PP1 phosphatase associated with one or more unknown regulatory subunits promotes dephosphorylation of S899, S911, and T912 in Pma1, in turn causing its self-inhibition.

Inhibition of the yeast Pma1 H^+^-ATPase in carbon-starved cells involves tight interaction between its catalytic (P) and C-terminal regulatory (R) domains. Upon glucose replenishment, this self-inhibitory interaction is disrupted in a manner involving R domain phosphorylation at residues S899 and S911-T912 (Fig 1A), this resulting in Pma1 activation [31,34,47]. A main conclusion of our study is that the long-sought kinases mediating tandem S911-T912 phosphorylation in Pma1 are the largely redundant Ptk1 and Ptk2 kinases. In glucose-grown cells, Ptk2 contributes more than Ptk1 to supporting Pma1 activity, and this tallies with a more active role of this kinase in S911-T912 phosphorylation. A lack of both Ptk kinases results in almost undetectable S911-T912 phosphorylation and no cell growth. These phenotypes are similar to those caused by the S911A-T912A double substitution in Pma1 [34,41]. Non-functionality of Pma1 caused by S911A-T912A or lack of Ptk1/2, both suppressed by the G648S substitution, likely results from constitutive self-inhibition of the H^+^-ATPase, although additional effects on its trafficking to the cell surface cannot be ruled out [34]. In a previous work, Ptk2 was shown to be essential to phosphorylation of S899 in the R domain of Pma1 [34], a modification whose impairment only partially impedes Pma1 activation by glucose. This indicates that Ptk1 cannot compensate for the loss of Ptk2 in carrying out this modification, in contrast to S911-T912 phosphorylation. As the *PTK1* and *PTK2* genes originate from whole-genome duplication (WGD) in a yeast ancestor [45], we propose that before WGD, a single Ptk kinase phosphorylated all three R-domain residues and that after WGD, the Ptk1 paralog lost the ability to target S899 (Fig 6A). The probable post-WGD functional divergence of Ptk1 and Ptk2 is also illustrated by the greater contribution of Ptk2 to S911-T912 phosphorylation and, as discussed below, by the different sensitivities of Ptk1 and Ptk2 to potential feedback control via TORC1. The Hrk1 kinase was reported to contribute to S911-T912 phosphorylation in Pma1, at least in cells grown on a medium at pH 4, treated or not with acetate [42]. In contrast, under the conditions of our experiments, Hrk1 alone does not significantly contribute to this phosphorylation as the latter was undetectable in the *ptk1Δ ptk2Δ* mutant in which Hrk1 is functional.

The recently solved structures of Pma1 hexamers purified from yeast [12] and *N*. *crassa* [11] revealed that the unphosphorylated R domain forms an alpha helix which interacts with the P domain of the same monomer and also with that of the neighboring monomer [11,12]. Interestingly, phosphorylation of S911-T912, or of equivalent residues in *N*. *crassa* Pma1, is reported mostly to disrupt trans R-P interactions [11,12]. Furthermore, phosphorylation of S901 in *N*. *crassa* Pma1, equivalent to S899 in yeast Pma1, is proposed to promote R-R contacts which could sequester together the untethered R domains in the central cavity of the ring-like Pma1 hexamer [12] (Fig 1A). Stimulation of Pma1 via R-domain phosphorylation might thus be most efficient when the H^+^-ATPase forms hexamers [12], although the 3D structure of yeast Pma1 suggests a potential additional contribution of S899 and S911 phosphorylation to destabilizing cis R-P interactions [11]. The size of the central cavity of the hexamer, as revealed by the solved structure of oligomerized Pma1, is also compatible with the view that Ptk kinases migrate into it to target residues S899, S911, and T912 [12].

Previous reports have shown the influence of single S899A, S911A, and T912A substitutions on Pma1 activity. The S899A substitution reduces the apparent affinity of Pma1 for ATP, without significantly perturbing cell growth [33,36]. The S911A substitution affects neither Pma1 activity nor cell growth, whereas T912A causes a strong reduction of the H^+^-ATPase Vmax and cell growth [33,41]. Intriguingly, although T912 phosphorylation seems more important in relieving self-inhibition of Pma1, phosphoproteomic analysis has revealed that glucose starvation elicits rapid reduction of S911 phosphorylation, whereas T912 phosphorylation is more stable [39]. This results in transient accumulation of Pma1 singly phosphorylated at T912. The rapid reduction of Pma1 activity nevertheless observed upon acute glucose starvation might be a consequence of rapid dephosphorylation of S911 and additional residues, for instance S899, combined with a drop in intracellular ATP. Subsequent dephosphorylation of T912 might then stabilize the Pma1 hexamer in its autoinhibited state, mainly via establishment of R-P trans-interactions.

Our results show that several proteins contribute to glucose starvation-elicited S911-T912 dephosphorylation in Pma1. One is the Snf1 kinase, known to be stimulated under carbon starvation [48]. Interestingly, previous studies have revealed that several Ptk2 residues, including S104-S105, undergo phosphorylation in glucose-deprived cells [49] and that these modifications are promoted by Snf1 [44]. This suggests a model where the Snf1 kinase phosphoinhibits Ptk2 in glucose-starved cells (Fig 6B). This putative control, however, must contribute only modestly to S911-T912 dephosphorylation, since the latter is only slightly impaired in the *snf1Δ* mutant. Our results show, instead, that the key actor of S911-T912 dephosphorylation upon glucose starvation is the Glc7 PP1 phosphatase (Fig 6B). We have highlighted this by studying a particular Glc7 mutant encoded by the *glc7-109* allele [51]. The corresponding Glc7(R260A) variant differs from others in that it causes hyperactivity of Pma1 [51]. The charged Arg260 residue altered in this variant is exposed at the surface of the protein and might thus mediate protein-protein interactions. We find that in the *glc7-109* mutant, dephosphorylation of S911-T912 induced by acute glucose starvation is largely impaired. Basal phosphorylation of S911-T912 in glucose-grown cells is also increased, suggesting that Glc7 also counteracts Ptk-dependent phosphorylation of S911-T912 when glucose is available. Intriguingly, it has been reported previously that upon acute glucose starvation, mutant cells expressing Glc7(R260A) display normal dephosphorylation of S911-T912 but impaired dephosphorylation of S899 [34]. The cause of the discrepancy between these and our results remains unclear. Of note, the fact that Pma1 is hyperactive in *glc7-109* cells [51] seems fully consistent with a role of Glc7 in S911-T912 dephosphorylation, given the greater contribution of S911-T912 than S899 phosphorylation to stimulating Pma1 activity.

The Glc7 PP1 phosphatase typically functions in tight conjunction with regulatory subunits [50]. In glucose-grown cells, for instance, Glc7 associates with Reg1 to dephosphorylate several proteins, including Snf1 [48]. This control is relieved upon glucose starvation. Yet the essential role of Glc7 in dephosphorylation of Pma1 S911-T912 illustrates that Glc7 is also active in glucose-starved cells, though toward other protein targets. Reg1 does not seem to play an important role in this Glc7 function, since a lack of Reg1 does not impair S911-T912 dephosphorylation in Pma1. We therefore propose that under glucose starvation, Glc7 associates with one or several other regulatory subunits to actively catalyze Pma1 S911-T912 dephosphorylation (Fig 6B) and potentially that of other proteins as well, as recently illustrated [59]. Our attempts to identify such Glc7 subunits by testing 22 single mutants were not successful, possibly because several of the tested proteins function redundantly with Glc7 to dephosphorylate S911-T912 in Pma1.

Upon H^+^ influx coupled to active nutrient uptake across the plasma membrane, TORC1 is activated via a pathway, not yet known, in which Pma1 plays an important role [19]. Our current model proposes that Pma1 stimulated by an H^+^ increase triggers molecular events culminating in rapid and transient TORC1 activation via the heterodimeric Gtr1/2 small GTPase complex. Stimulation of Pma1 activity under acidic conditions was reported many years ago and correlates with increased affinity of the H^+^ pump for ATP [60–62]. The molecular mechanisms underlying this stimulation remain only partially known. According to previous reports, Pma1 stimulation in acidic environments coincides with a detectable conformational change [63], potential destabilization of salt bridges between the R and P domains [11], and increased phosphorylation, via the Hrk1 kinase, of S911-T912 and additional residues in the A, N, and P domains [42]. Cytosol acidification also likely favors titration of D730 residue, the first H^+^ acceptor in the translocation path of Pma1, the pKa of which is proposed to change according to the activity state of the H^+^ pump [11]. In our experiments, we found H^+^-coupled uptake of β-alanine, which cannot be used as nitrogen source [19], to induce a significant increase in both Ptk1- and Ptk2-dependent S911-T912 phosphorylation. This increased phosphorylation is not a consequence of TORC1 activation as it is also visible in a mutant lacking the Gtr1/2 small GTPases (S8 Fig). These observations suggest that in glucose-grown cells, an H^+^ increase can potentially contribute to Pma1 stimulation through relief from some residual autoinhibition. It also raises the possibility that Ptk kinases become more active upon H^+^ influx in the cytosol. Interestingly, the increase in S911-T912 phosphorylation observed upon H^+^-coupled amino-acid uptake seems less pronounced in a mutant lacking Hrk1 (S1C Fig). Yet this kinase, reported to be active under acidic conditions [42], is unable to sustain significant S911-T912 phosphorylation in a *ptk1Δ ptk2Δ* mutant. This suggests that Hrk1 might stimulate Ptk1 and Ptk2 in response to H^+^ increase. On the other hand, C-terminally truncated Pma1 variants still display higher ATPase activity at acidic pH [11,64], suggesting that Pma1 can also be stimulated by acidic pH independently of neutralization of its autoinhibitory R domain. Consistently, we found that TORC1 is still activated upon H^+^-coupled β-alanine uptake into cells expressing the truncated Pma1(Δ890–918) mutant. This shows that the R domain of Pma1 is not required for TORC1 activation upon H^+^ influx. We have nevertheless observed that in cells lacking the Ptk2 kinase, TORC1 activation upon H^+^-coupled amino-acid uptake is reduced vs. the wild-type, an effect not due to reduced uptake of the amino acid. One model that might account for this observation is that stimulation of Pma1 by an H^+^ increase is hindered by partial clamping caused by reduced phosphorylation of the R domain. It is also possible that the R domain, when tethered to the cytosolic P domain, interferes with certain interactions between Pma1 and putative factors involved in the early steps of H^+^-responsive TORC1 activation. Additional work is clearly needed to elucidate how Pma1 is stimulated by an H^+^ increase and how it then promotes TORC1 activation.

According to previous reports, phosphorylation of upstream TORC1 regulators is negatively regulated in a manner dependent on TORC1 itself [53–55]. Such feedback controls are assumed to avoid TORC1 overactivation. Excess TORC1 activity can indeed be detrimental to cell growth, especially under non-optimal growth conditions normally associated with only moderate TORC1 activation [54,65]. In this study, we have likewise obtained evidence that TORC1 might feedback-control Pma1 by modulating its S911-T912 phosphorylation. Specifically, TORC1 inhibition by rapamycin or nitrogen starvation results in increased Ptk-dependent phosphorylation of S911-T912. Analysis of cells expressing a single Ptk kinase indicates, furthermore, that stimulation of S911-T912 phosphorylation upon TORC1 inhibition is more pronounced in Ptk1- vs. Ptk2 cells. This further illustrates the functional divergence of the two Ptk kinase paralogs. In further support of the view that TORC1 negatively feedback-controls Pma1 phosphorylation, we found this phosphorylation to decrease a few minutes after β-alanine-uptake-triggered TORC1 activation. These observations suggest the following model (Fig 5D): in nutrient-starved cells, H^+^ increase coupled to uptake of replenishing nutrients first provokes an Hrk1- and Ptk-dependent rise in Pma1 phosphorylation at S911-T912 and a parallel TORC1 activation. Once TORC1 has been stimulated, it favors downregulation of Pma1 activity via reduction of its S911-T912 phosphorylation. This model is compatible with studies of the phosphoproteome in rapamycin-treated cells, revealing significant changes in the phosphorylation of residues in the Ptk1, Ptk2 and Hrk1 kinases [57,58]. According to another report, furthermore, TORC1 up-regulates Glc7 PP1 phosphatase activity [66]. Hence, TORC1 stimulated by an H^+^ influx might possibly modulate the functions of both the Glc7 phosphatase and the Hrk1 and Ptk kinases, thus causing reduced Pma1 S911-T912 phosphorylation. Further investigation is needed to specify which branch of TORC1 controls S911-T912 phosphorylation in Pma1. Previous works reported that the Sit4 phosphatase positively controls Pma1 activity and protein levels [67,68], suggesting that putative TORC1-mediated control of Ptk kinases involves the Tap41/42-Sit4 branch. In support of this view, phosphorylation of Ptk2 is reported to be reduced in the *tap42* mutant [57]. Of note, another study reported that TORC1 exerts a positive effect on Pma1 activity [67] but this conclusion is based on cell treatments inactivating TORC1 for several hours that likely hampered cell metabolism and thus potentially Pma1 activity.

Importantly, another condition causing a cytosolic H^+^ increase and Pma1-dependent TORC1 activation is acute inhibition of the vacuolar V-ATPase complex [1,19]. This inhibition, furthermore, is reported to promote ubiquitin-dependent endocytosis of Pma1 [69]. This downregulation requires the Glc7 phosphatase acting together with Reg1, and also the calcium-responsive phosphatase calcineurin [70]. These observations raise the interesting possibility that the negative control of Pma1 proposed here to occur upon TORC1 activation might act not only on the activity the H^+^-ATPase but also on its stability at the PM. In this context, a model has been discussed where Pma1 with a dephosphorylated R domain might be more prone to ubiquitination and thus endocytosis [70]. Whatever the exact mechanisms involved, Pma1 downregulation upon H^+^ increase is likely regulated tightly so as to avoid excessive TORC1 stimulation and at the same time to retain sufficient H^+^-ATPase activity at the PM to expel excess H^+^ and preserve cell viability. Consistently with the view that this intricate control of Pma1 is important for cell homeostasis, inhibition of the V-ATPase is particularly detrimental to cells impaired in Pma1 endocytosis [70]. Other promising directions for future studies are thus to determine whether this phenotype is due to TORC1 dysregulation and to dissect the molecular details of the probable crosstalk between TORC1 and the multiple factors involved in Pma1 regulation.

## Materials and methods

### Yeast strains and growth conditions

The *Saccharomyces cerevisiae* strains used in this study (Table 1) derive from the wild type Σ1278b, except in experiments of Fig 3D and S7 Fig (strains KT1112 and KT4078) and S5 Fig (strains BY4742 and derivates). Leucine and uracil auxotrophies were complemented by plasmids. Mutant strain ES193 was isolated by CRISPR/Cas9 as previously described [71]. The plasmids used in this study are listed in Table 2. The plasmid-borne *PMA1* alleles were expressed under the natural *PMA1* gene’s promoter. Cells were grown at 29°C on a minimal medium buffered at pH 6.1 [72] and containing glucose, galactose, or glycerol (each at 3% w/v) as carbon source and (NH_4_)_2_SO_4_ (10 mM) or proline (10 mM) as nitrogen source. When specified, synthetic Drop-out Medium (Sigma-Aldrich, Y1501) was provided in addition to (NH_4_)_2_SO_4_ as nitrogen source. For comparing the growth rates of strains and their sensitivities to tetramethylammonium (Sigma-Aldrich, Product n° T3411), culture samples diluted to an OD_660_ (optical density at 660 nm) of 0.02 were used to fill the wells of a Greiner 24-well microplate, which was then incubated at 29°C with shaking in a SYNERGY multi-mode reader (BioTek Instruments). The OD_660_ values measured for 24 hours were then analyzed in order to calculate the minimal doubling times. For growth tests on solid medium, cell suspensions diluted to OD_660_ = 0.1 were spread at the surface of media containing 1% agarose, and the Petri dishes were incubated at 29°C for 2–4 days.

**Table 1:** Strains used in this study.

Strain	Genotype	Source
23344c	*ura3*	Lab collection
CF148	*ptk1Δ ura3*	This study
CF152	*ptk2Δ ura3*	This study
OS26-1	*gtr1Δ gtr2Δ ura3*	[19]
27033d	*ura3 leu2*	Lab collection
CF055	*ptk2Δ ura3 leu2*	This study
CF081	*hrk1Δ ura3 leu2*	This study
CF088	*ptk2Δ hrk1Δ ura3 leu2*	This study
JX023	*pGAL1-PMA1 pma2Δ ura3 leu2*	[19]
43160c	*pGAL1-PMA1 pma2Δ ptk2Δ ura3 leu2*	This study
CF166	*pGAL1-PMA1 pma2Δ ptk2Δ ura3 leu2*	This study
ES193	*PMA1Δ(890–918) ura3 leu2*	This study
43458b	*PMA1Δ(890–918) ptk1Δ ptk2Δ ura3 leu2*	This study
JB002	*pGAL1-PMA1(Δ890–918) pma2Δ ptk1Δ ptk2Δ ura3 leu2*	This study
MEV30	*snf1Δ ura3*	This study
CF199	*ptk1Δ snf1Δ ura3*	This study
KT1112	*ura3 leu2 his3*	[73]
KT4078	*glc7-109 ura3 leu2 his3*	[51]
CF259	*reg1Δ ura3*	This study

**Table 2:** Plasmids used in this study.

Plasmid	Description	Source
pFL38	CEN-ARS (URA3)	[74]
pFL36	CEN-ARS (LEU2)	[74]
pPS15-P1	CEN-ARS PMA1 (LEU2)	[75]
pS899A	CEN-ARS PMA1-S899A (LEU2)	[41]
pS899D	CEN-ARS PMA1-S899D (LEU2)	[41]
pS911A	CEN-ARS PMA1-S911A (LEU2)	[41]
pT912A	CEN-ARS PMA1-T912A (LEU2)	[41]
pS911A-T912A	CEN-ARS PMA1-S911AT912A (LEU2)	[41]
pCJ647	CEN-ARS PMA1(Δ890–918) (LEU2)	This study
pG648S	CEN-ARS PMA1-G648S (LEU2)	[32]
pJB001	CEN-ARS PTK1 (URA3)	This study
pCJ652	CEN-ARS PTK2 (URA3)	This study
pNG003	CEN-ARS PMA1-G648S-S911A-T912A (LEU2)	This study
pJYS20	CEN-ARS (HIS3-LEU2)	Lab collection

### Radiolabeled β-alanine uptake measurements

Accumulation of [^14^C]-labeled β-alanine (Hartmann analytic) was measured at the indicated time points as previously described [76,77]. Data points represent averages of biological replicates and error bars represent standard deviations (SD).

### Measurements of acidification of external medium

A previously published protocol [78] was applied with minor modifications. Yeast cells were grown on glucose NH_4_^+^ medium to OD_660_ ~0.2 and 100 ml culture was harvested, washed three times with 10 ml cold water, resuspended in a vial containing 25 ml of 0.2 mM MES buffer at pH 6.1 (KOH), and placed in a shaking incubator at 29°C. A pH electrode was then used to record the external pH for 20 min (time needed to stabilize the external pH) prior to addition of glucose (200 mM) and KCl (20 mM) (time 0 of the experiment). The pH was then recorded for another 10 min. Data points represent averages of at least three biological replicates and error bars represent standard deviations (SD).

### Cell extract preparation and immunoblot analyses

For most Sch9 and Pma1 immunoblots, cell lysates were prepared as previously described [79], except for those shown in Figs 4C, 4E, 4G, 5E, S1C, S6, S7 and S8 Figs which were prepared by an alternative method [80]. Cell lysates were subjected to SDS-PAGE, transferred to a nitrocellulose blotting membrane (Amersham product: 10600007), and probed with the following primary antibodies: rabbit anti-phospho-S911-T912-Pma1 [34], rabbit anti-C-tail-Pma1 [46] or anti-SpPma1 (Pma1 of *Schizosacchromyces pombe*) [81] to detect total Pma1, rabbit anti-yeast 3-phosphoglycerate kinase (PGK) (Invitrogen, Ref: 459250), mouse-anti-actin (Sigma, product: A2066), rabbit anti-phospho-Thr^737^-Sch9 [19], and anti-Sch9 [82] to detect total Sch9. Primary antibodies were detected with horseradish-peroxidase-conjugated anti-rabbit (Cytiva, NA934) or anti-mouse (Cytiva, NA931) immunoglobulin G secondary antibodies by enhanced chemiluminescence (Roche; catalog number 12 015 196 001). Each immunoblotting experiment was done at least twice and a representative experiment is presented.

### Reproducibility of experiments and statistics

Experiments illustrated in main figures were carried out two to five times. The significance of differences between the results of biological replicates was determined using Student’s two-tailed *t*-test. A *P* value of less than 0.05 was the significance criterion. Data and statistical analysis are presented in S1 Table.

## Supporting information

S1 Fig Influence of the Hrk1 kinase on Pma1 phosphorylation at S911-T912. (A) Immunoblot analysis of Pma1 and its phosphorylation at S911-T912 in lysates prepared from wild-type *(w-t)*, *hrk1Δ*, *ptk2Δ* and *hrk1Δ* *ptk2Δ* mutant cells growing exponentially in minimal glucose medium (+), transferred for 10 minutes to glucose-free medium (-), and replenished with glucose for 10 or 30 min. (B) Equivalent uptake of [^14^C]-β-alanine in wild-type *(w-t)* and *hrk1Δ* cells. The cells were initially grown to exponential phase in glucose NH_4_^+^ medium before being transferred for two hours to the same medium except that it lacked nitrogen. The labeled amino acid was then added (time 0 min) at a final concentration of 0.25 mM (*w-t*) or 0.2 mM (*hrk1Δ*). (C) *Left*. Immunoblot analysis of Pma1 and its phosphorylation at S911-T912 in lysates prepared from wild-type *(w-t)* and *hrk1Δ* cells as in B. *Right*. Quantification of relative pS911-pT912 phosphorylation (vs. total Pma1) seven minutes after β-alanine addition.
(PDF)

S2 Fig Influence of the Ptk1 kinase on Pma1 H^+^ export activity and phosphorylation at S911-T912. (A) pH variations normalized vs. OD_660_ measured upon glucose addition to glucose-starved wild-type *(w-t)* and *ptk1Δ* mutant cells. (B) Immunoblot analysis of Pma1 and its phosphorylation at S911-T912 in lysates prepared from cells as in A growing exponentially in minimal glucose medium (+), transferred for 10 minutes to glucose-free medium (-), and replenished with glucose for 10 or 30 min. (C) Relative minimal doubling times (% of untreated *w-t*) of wild-type cells (*w-t*), *ptk1Δ*, and *ptk2Δ* mutant cells during their growth in liquid minimal glucose medium supplemented with TMA (500 μM). Bars represent averages ± standard deviation (n = 3). *P* values obtained from the two-tailed paired *t* test are indicated.
(PDF)

S3 Fig Analysis of haploid spores after meiosis of the diploid obtained by crossing the *ptk1Δ* and *ptk2Δ* mutants. The indicated genotypes of haploid spores were deduced from resistances to different antibiotics associated with *PTK1* and *PTK2* deletion.
(PDF)

S4 Fig Influence of S899A and S899D substitutions on Ptk1- and Ptk2-dependent phosphorylation of Pma1 at S911-T912. Immunoblot analysis of Pma1 and its phosphorylation at S911-T912 in lysates prepared from cells growing exponentially on minimal galactose medium before (-) and 30 min after (+) glucose addition. The strains were *pGAL1-PMA1Δ(890–918) pma2Δ* *ptk1Δ* *ptk2Δ* expressing from plasmids the indicated *PMA1* allele and *PTK* gene.
(PDF)

S5 Fig Influence of different Glc7 regulatory subunits on Pma1 de-phosphorylation upon glucose starvation. (A, B, C, D) Immunoblot analysis of Pma1 and its phosphorylation at S911-T912 in lysates prepared from wild-type cells (*w-t*, BY4742) and from the indicated deletion mutants from the Euroscarf collection, harvested during exponential growth in minimal glucose medium (0 min) and after transfer for 4 minutes to the same medium except that no carbon source was available. The medium was supplemented with NH_4_^+^ (20 mM), yeast synthetic Drop-out, and uracil (0.1mM).
(PDF)

S6 Fig TORC1 activation upon β-alanine uptake into nitrogen-starved cells. (A) Immunoblot analysis of Sch9 and its phosphorylation at T737 in lysates prepared from *pGAL1-PMA1 pma2Δ* cells expressing *PMA1* from a plasmid. The cells were initially grown to exponential phase in a glucose NH_4_^+^ medium (+ N) before being transferred for two hours to the same medium except that it lacked nitrogen (0 min). β-alanine (0.2 mM) was then added for the indicated times. (B) Equivalent uptake of [^14^C]-β-alanine in wild-type *(w-t)* and *gtr1Δ* *gtr2Δ* cells. The cells were treated as in A and the labeled amino acid was added (time 0 min) at a final concentration of 0.5 mM. (C) Immunoblot analysis of Sch9 and its phosphorylation at T737 in lysates prepared from wild-type *(w-t)* and *gtr1Δ* *gtr2Δ* cells treated as in B.
(PDF)

S7 Fig Influence of *glc7-109* mutation on TORC1 activation upon H^+^-coupled amino-acid uptake. (A) Equivalent uptake of [^14^C]-β-alanine in wild-type *(w-t) and glc7-109* cells. The cells were initially grown to exponential phase in glucose NH_4_^+^ medium before being transferred for two hours to the same medium except that it lacked nitrogen. The labeled amino acid was then added (time 0 min) at a final concentration of 0.5 mM. (B) Immunoblot analysis of Sch9 and its phosphorylation at T737 in lysates prepared from cells as in A.
(PDF)

S8 Fig Lack of Gtr1 Gtr2 does not impede increased phosphorylation of Pma1 at S911-T912 upon H^+^-coupled amino-acid uptake. Immunoblot analysis of Pma1 and its phosphorylation at S911-T912 in lysates prepared from wild-type (*w-t*) and *gtr1Δ* *gtr2Δ* mutant cells growing exponentially in glucose NH_4_^+^ medium (+N), transferred for 2 hours to nitrogen-free medium (time 0), and incubated for the indicated time with β-alanine (0.5 mM). The amino acid was incorporated at equivalent rates in the two strains (S6 Fig).
(PDF)

S1 Table Quantification of data and statistical analysis. (XLSX)
