# Supplementary figures and images for: Phosphoregulation of the yeast Pma1 H+-ATPase autoinhibitory domain involves the Ptk1/2 kinases and the Glc7 PP1 phosphatase and is under TORC1 control
Source: PLoS Genet. 2024 Jan 16;20(1):e1011121. doi: 10.1371/journal.pgen.1011121 (PMC10817110; doi:10.1371/journal.pgen.1011121)

**A**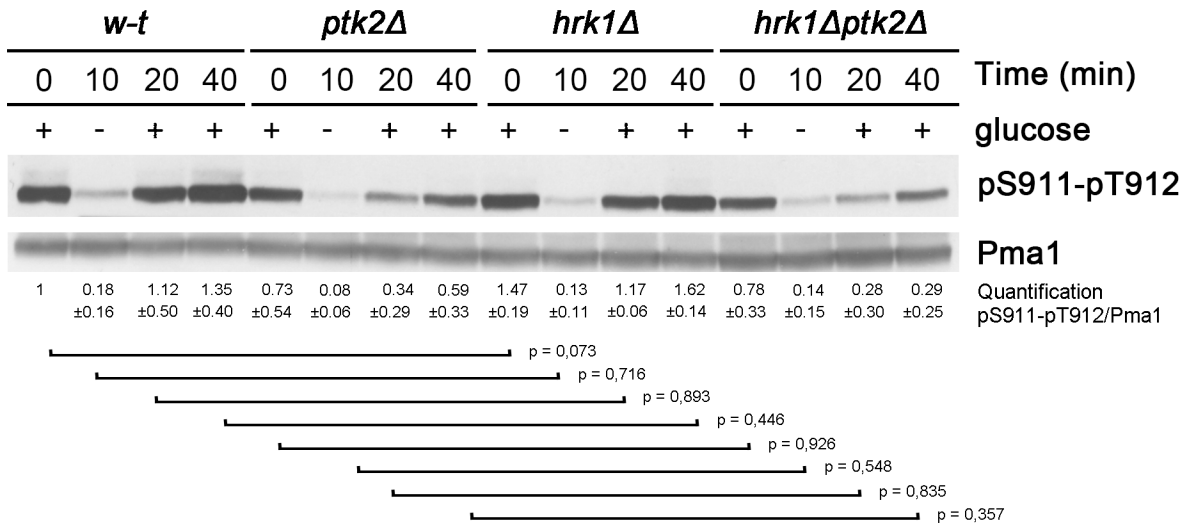**B**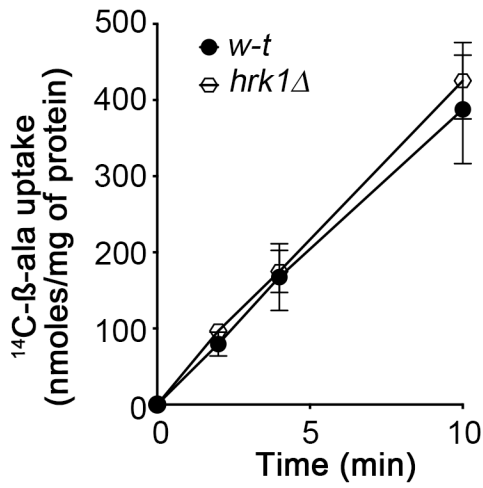**C**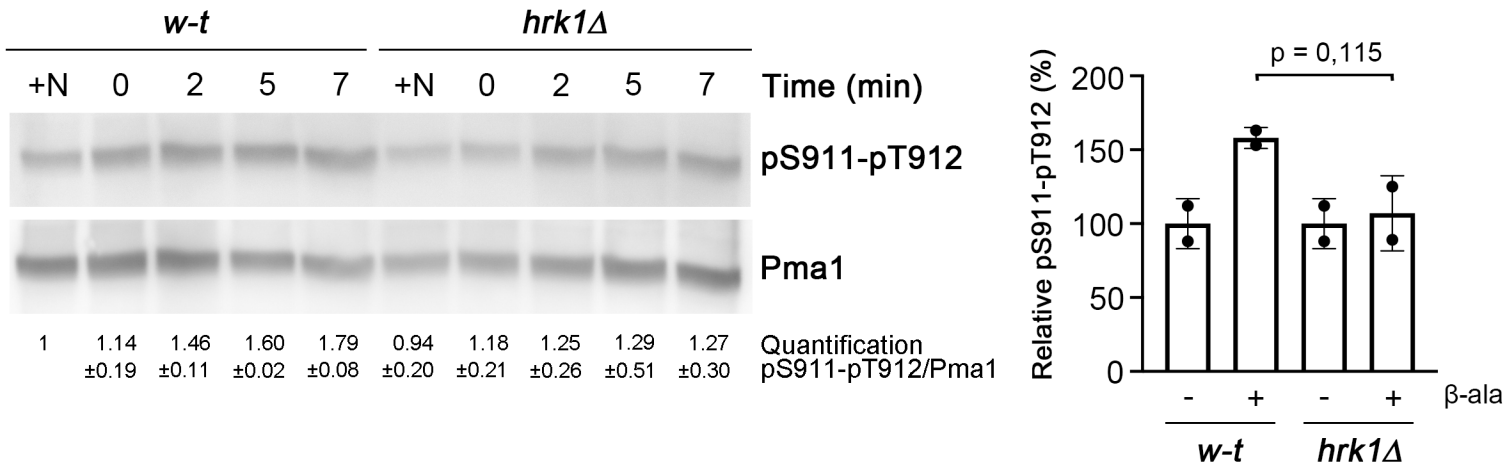

Supplement: S1 Fig — (A) Immunoblot analysis of Pma1 and its phosphorylation at S911-T912 in lysates prepared from wild-type (w-t), hrk1Δ, ptk2Δ and hrk1Δ ptk2Δ mutant cells growing exponentially in minimal glucose medium (+), transferred for 10 minutes to glucose-free medium (-), and replenished with glucose for 10 or 30 min. (B) Equivalent uptake of [14C]-β-alanine in wild-type (w-t) and hrk1Δ cells. The cells were initially grown to exponential phase in glucose NH4+ medium before being transferred for two hours to the same medium except that it lacked nitrogen. The labeled amino acid was then added (time 0 min) at a final concentration of 0.25 mM (w-t) or 0.2 mM (hrk1Δ). (C) Left. Immunoblot analysis of Pma1 and its phosphorylation at S911-T912 in lysates prepared from wild-type (w-t) and hrk1Δ cells as in B. Right. Quantification of relative pS911-pT912 phosphorylation (vs. total Pma1) seven minutes after β-alanine addition. (PDF) [file pgen.1011121.s001.pdf]

A

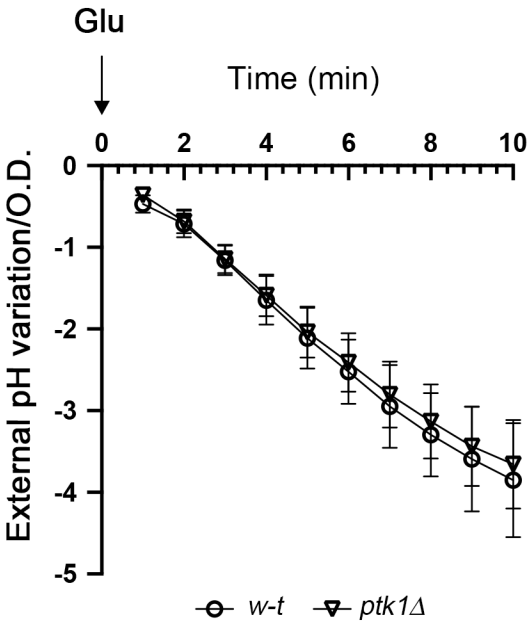

B

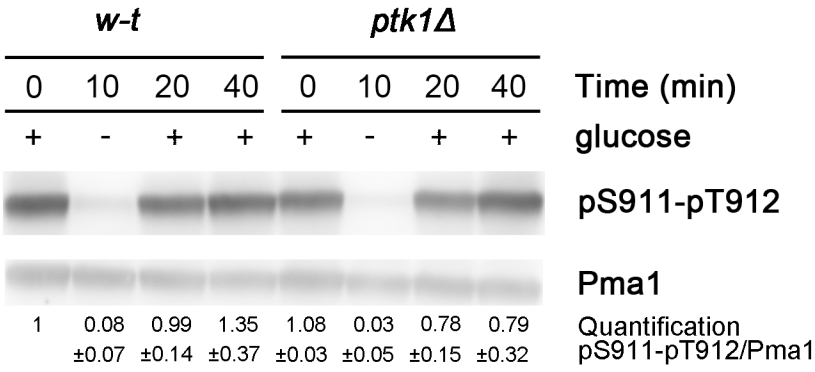

C

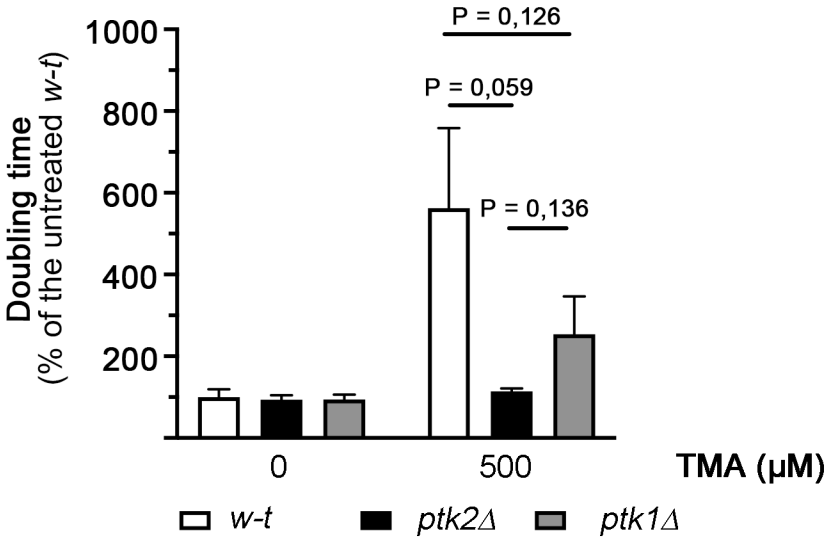

Supplement: S2 Fig — (A) pH variations normalized vs. OD660 measured upon glucose addition to glucose-starved wild-type (w-t) and ptk1Δ mutant cells. (B) Immunoblot analysis of Pma1 and its phosphorylation at S911-T912 in lysates prepared from cells as in A growing exponentially in minimal glucose medium (+), transferred for 10 minutes to glucose-free medium (-), and replenished with glucose for 10 or 30 min. (C) Relative minimal doubling times (% of untreated w-t) of wild-type cells (w-t), ptk1Δ, and ptk2Δ mutant cells during their growth in liquid minimal glucose medium supplemented with TMA (500 μM). Bars represent averages ± standard deviation (n = 3). P values obtained from the two-tailed paired t test are indicated. (PDF) [file pgen.1011121.s002.pdf]

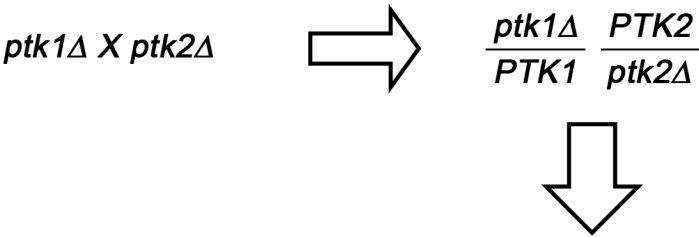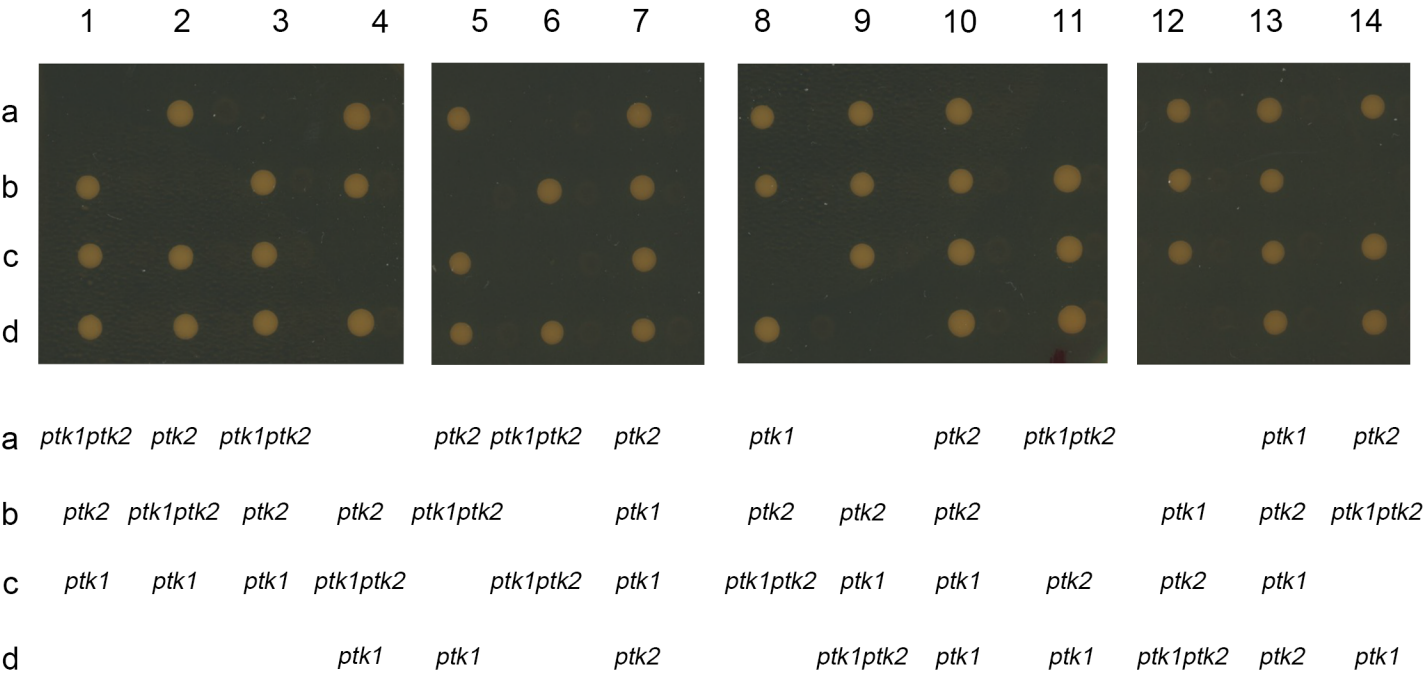

Supplement: S3 Fig — The indicated genotypes of haploid spores were deduced from resistances to different antibiotics associated with PTK1 and PTK2 deletion. (PDF) [file pgen.1011121.s003.pdf]

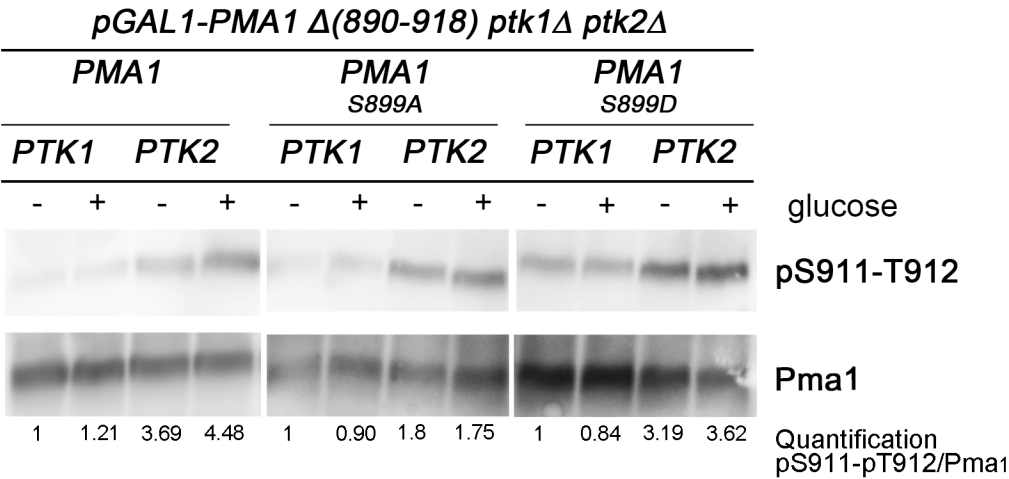

Supplement: S4 Fig — Immunoblot analysis of Pma1 and its phosphorylation at S911-T912 in lysates prepared from cells growing exponentially on minimal galactose medium before (-) and 30 min after (+) glucose addition. The strains were pGAL1-PMA1Δ(890–918) pma2Δ ptk1Δ ptk2Δ expressing from plasmids the indicated PMA1 allele and PTK gene. (PDF) [file pgen.1011121.s004.pdf]

A

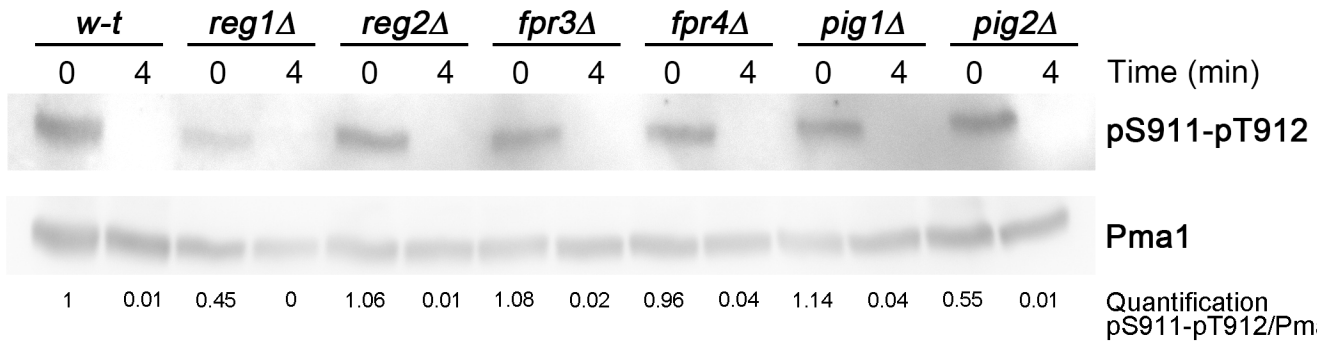

B

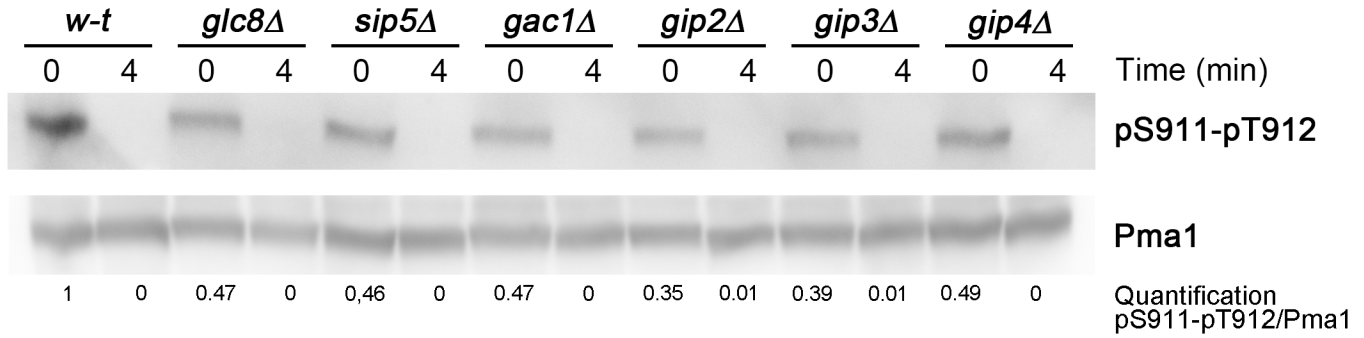

C

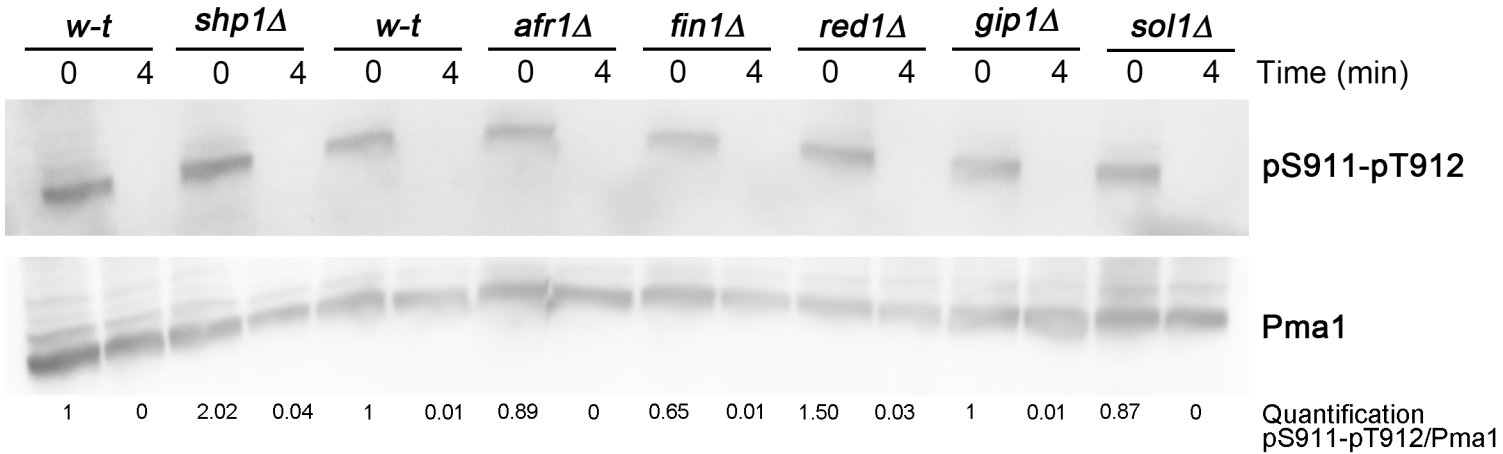

D

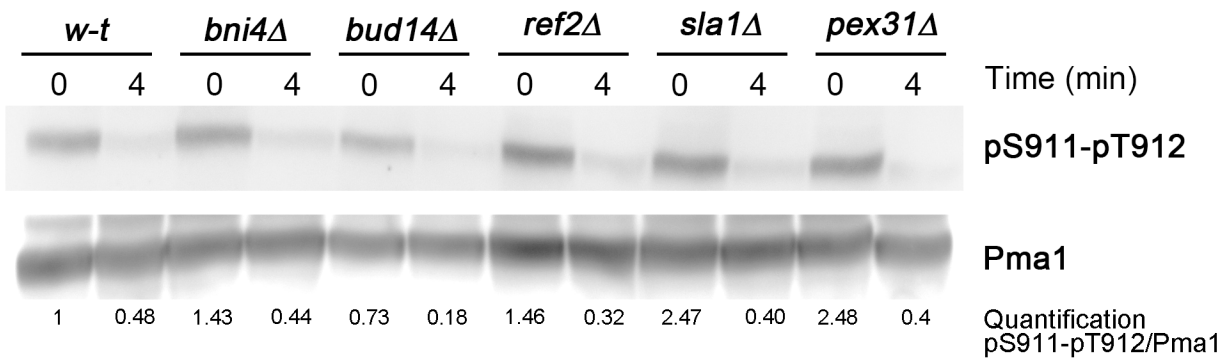

Supplement: S5 Fig — (A, B, C, D) Immunoblot analysis of Pma1 and its phosphorylation at S911-T912 in lysates prepared from wild-type cells (w-t, BY4742) and from the indicated deletion mutants from the Euroscarf collection, harvested during exponential growth in minimal glucose medium (0 min) and after transfer for 4 minutes to the same medium except that no carbon source was available. The medium was supplemented with NH4+ (20 mM), yeast synthetic Drop-out, and uracil (0.1mM). (PDF) [file pgen.1011121.s005.pdf]

**A**

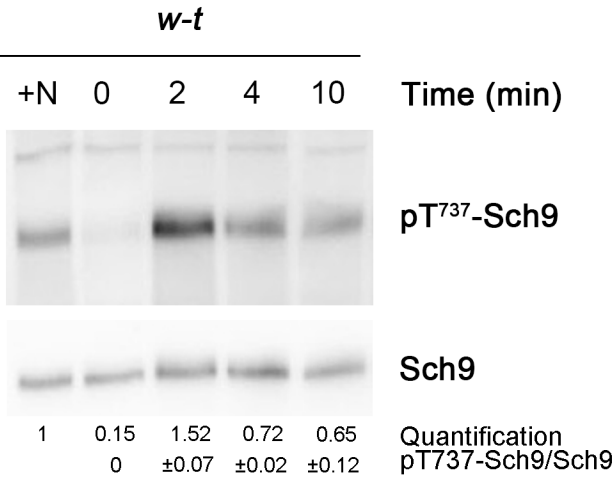

**B**

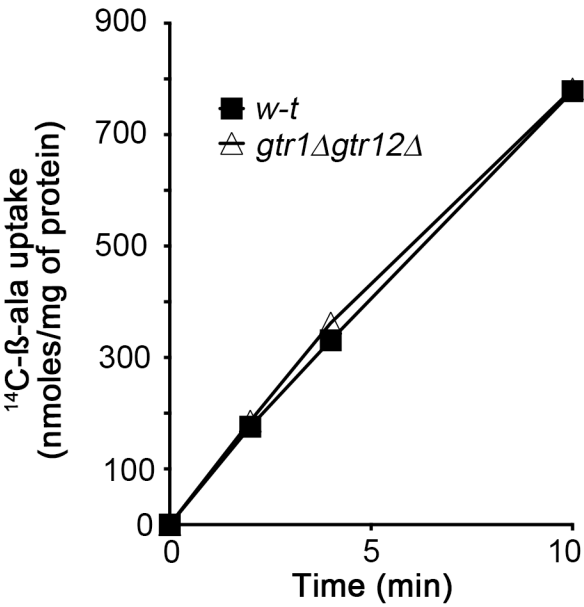

**C**

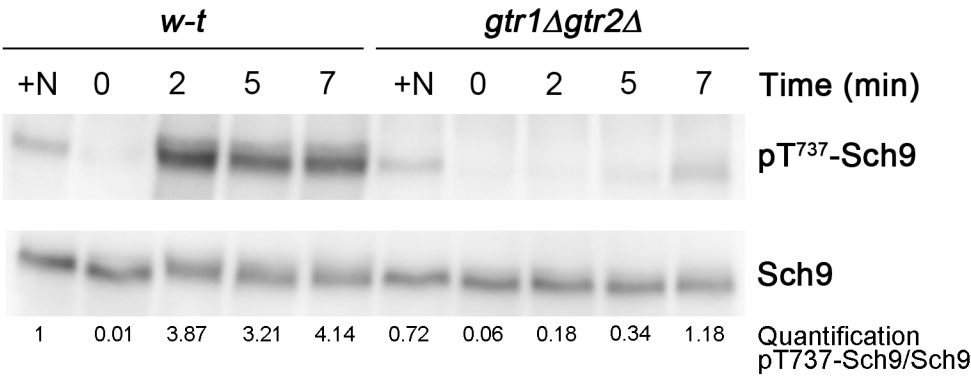

Supplement: S6 Fig — (A) Immunoblot analysis of Sch9 and its phosphorylation at T737 in lysates prepared from pGAL1-PMA1 pma2Δ cells expressing PMA1 from a plasmid. The cells were initially grown to exponential phase in a glucose NH4+ medium (+ N) before being transferred for two hours to the same medium except that it lacked nitrogen (0 min). β-alanine (0.2 mM) was then added for the indicated times. (B) Equivalent uptake of [14C]-β-alanine in wild-type (w-t) and gtr1Δ gtr2Δ cells. The cells were treated as in A and the labeled amino acid was added (time 0 min) at a final concentration of 0.5 mM. (C) Immunoblot analysis of Sch9 and its phosphorylation at T737 in lysates prepared from wild-type (w-t) and gtr1Δ gtr2Δ cells treated as in B. (PDF) [file pgen.1011121.s006.pdf]

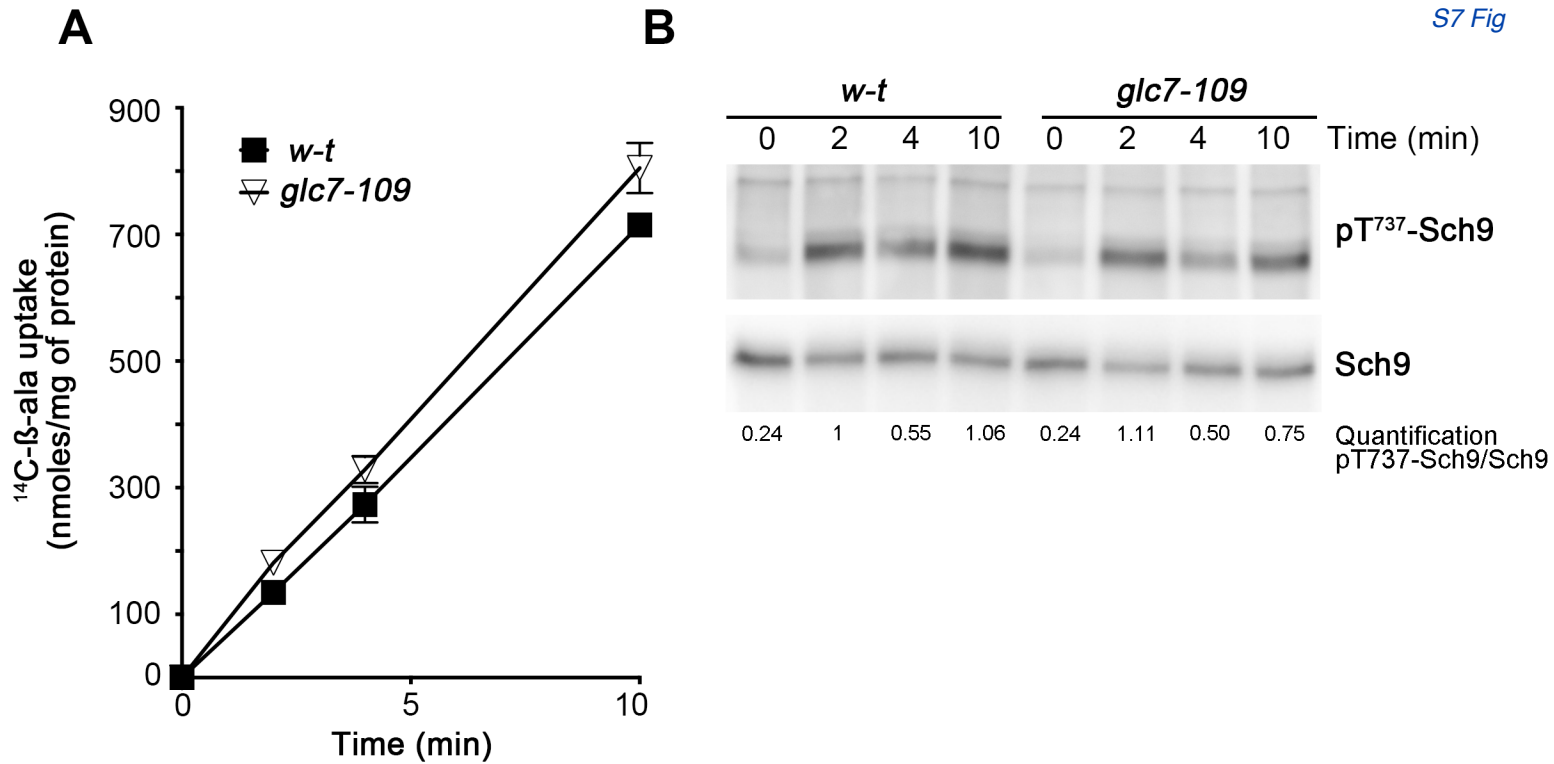

Supplement: S7 Fig — (A) Equivalent uptake of [14C]-β-alanine in wild-type (w-t) and glc7-109 cells. The cells were initially grown to exponential phase in glucose NH4+ medium before being transferred for two hours to the same medium except that it lacked nitrogen. The labeled amino acid was then added (time 0 min) at a final concentration of 0.5 mM. (B) Immunoblot analysis of Sch9 and its phosphorylation at T737 in lysates prepared from cells as in A. (PDF) [file pgen.1011121.s007.pdf]

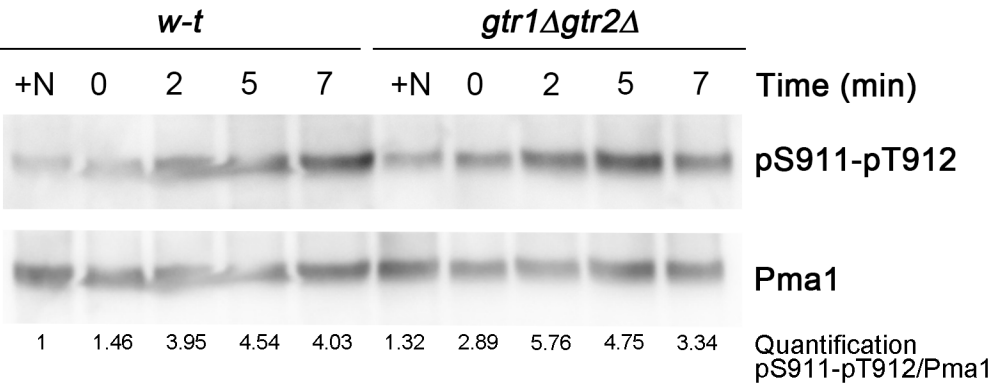

Supplement: S8 Fig — Immunoblot analysis of Pma1 and its phosphorylation at S911-T912 in lysates prepared from wild-type (w-t) and gtr1Δ gtr2Δ mutant cells growing exponentially in glucose NH4+ medium (+N), transferred for 2 hours to nitrogen-free medium (time 0), and incubated for the indicated time with β-alanine (0.5 mM). The amino acid was incorporated at equivalent rates in the two strains (S6 Fig). (PDF) [file pgen.1011121.s008.pdf]
